# Design of Bacterial Strain-Specific qPCR Assays Using NGS Data and Publicly Available Resources and Its Application to Track Biocontrol Strains

**DOI:** 10.3389/fmicb.2020.00208

**Published:** 2020-03-10

**Authors:** Iker Hernández, Clara Sant, Raquel Martínez, Carolina Fernández

**Affiliations:** Futureco Bioscience S.A., Barcelona, Spain

**Keywords:** bacterial strain-specific, biocontrol, marker, NGS, qPCR, risk assessment

## Abstract

Biological control is emerging as a feasible alternative to chemical pesticides in agriculture. Measuring the microbial biocontrol agent (mBCA) populations in the environment is essential for an accurate environmental and health risk assessment and for optimizing the usage of an mBCA-based plant protection product. We hereby show a workflow to obtain a large number of qPCR markers suitable for robust strain-specific quantification. The workflow starts from whole genome sequencing data and consists of four stages: (i) identifying the strain-specific sequences, (ii) designing specific primer/probe sets for qPCR, and (iii) empirically verifying the performance of the assays. The first two stages involve exclusively computer work, but they are intended for researchers with little or no bioinformatic background: Only a knowledge of the BLAST suite tools and work with spreadsheets are required; a familiarity with the Galaxy environment and next-generation sequencing concepts are strongly advised. All bioinformatic work can be implemented using publicly available resources and a regular desktop computer (no matter the operating system) connected to the Internet. The workflow was tested with five bacterial strains from four different genera under development as mBCAs and yielded thousands of candidate markers and a triplex qPCR assay for each candidate mBCA. The qPCR assays were successfully tested in soils of different natures, water from different sources, and with samples from different plant tissues. The mBCA detection limits and population dynamics in the different matrices are similar to those in qPCR assays designed by other means. In summary, a new accessible, cost-effective, and robust workflow to obtain a large number of strain-specific qPCR markers is presented.

## Introduction

Identification and quantification of bacteria at strain level are cornerstones in several fields such as medicine, microbiology, food science and technology, aquaculture, and plant biology (Marx, 2016). The most common techniques to monitor bacteria at strain level are dilution-plate counting and PCR-derived methods. Other techniques, such as Raman spectroscopy and metabolite profiling by mass spectrometry, with their derivatives (Pavlicek et al., 2017; Sauget et al., 2017), have been also used, but their routine use is limited due to the need of highly specialized equipment. Dilution-plate counting is the most straightforward procedure, but it is only useful when selective media are available or when the target strain has unique and conspicuous phenotypic traits, and it is rarely specific at strain level (Koch, 2010). In addition, plate counting only accounts for viable cells, thereby excluding inviable and viable but non-culturable (VBNC) cells (Koch, 2010). PCR is highly specific so long as the primers anneal only to DNA sequences that are specific of the intended target strain. These DNA sequences are most often identified by DNA fingerprinting techniques such as RAPD (Random Amplified Polymorphic DNA) or AFLP (Amplification Fragment Length Polymorphism) followed by sequence characterization (Hermosa et al., 2001; Dauch et al., 2003; Felici et al., 2008; Xiang et al., 2010; Perez et al., 2014). Combining DNA fingerprinting and real-time quantitative PCR (qPCR) provides a powerful tool to identify and quantify bacteria at strain level, and this has made it a widespread method to monitor bacterial strains when dilution-plate counting is not feasible. PCR targets DNA sequences that may be present in viable cells, VBNC, or cell debris, but further refinement using viability PCR (vPCR) is gaining importance to distinguish viable from other states in the target bacterial populations (Nogva et al., 2003; Elizaquivel et al., 2014).

Despite of the abundant literature reporting bacterial strain monitoring methods by PCR using sequence-characterized DNA fragments obtained by RAPD, AFLP, and other DNA fingerprinting methods, these procedures are laborious and time- and resource-consuming and usually produce very few useful marker sequences, which may become helpless as new strains of closely related taxa are discovered and characterized (Hermosa et al., 2001; Dauch et al., 2003; Felici et al., 2008; Xiang et al., 2010; Perez et al., 2014). Next-generation sequencing (NGS) technologies offer the possibility to obtain DNA sequence information from bacterial strains of interest in a faster and more cost-effective manner as compared to the aforementioned methods (Marx, 2016). In addition, some NGS platforms allow tuning the amount of data to be generated to the experiment needs, thereby rendering sequencing even more flexible and cost-effective. Short read NGS platforms are the most extended ones due to their accuracy and lower costs (Steinbock and Radenovic, 2015), although the use of long read platforms is growing because long reads are computationally more convenient for the assembly of bacterial genomes *de novo* (Koren and Phillippy, 2015) and recent developments have considerably reduced their error rate (Wenger et al., 2019).

Owing to the nature and size of NGS data, most analysis tools have been developed to perform a discrete task within a workflow, which is to be defined by the user. Thus, one has tools for raw data quality control, read mapping, assembly, scaffolding, counting, file format grooming, etc. Most of these tools are implemented through the command line and thus require the user to be familiar with advanced computational methods as well as specialized computing resources. This turns NGS data analysis into the bottleneck of biological experiments when researchers lack bioinformatic expertise. However, there is growing awareness among developers that their tools are used by non-bioinformaticians on their desktop computers, thereby limiting the reach of their contributions to the field of deep sequencing data analysis. In consequence, bioinformatic tools tend to offer user-friendly versions fueled by powerful remote computing resources. Still, as mentioned before, the user must define the workflow that tackles their research objectives and suits their needs.

In this manuscript, we present a workflow to obtain a large number of PCR markers suitable for robust strain-specific quantification. This workflow starts from raw NGS data and uses publicly available and user-friendly resources. Furthermore, the workflow is applied to five bacterial strains under development as microbial biocontrol agents (mBCAs) in several sample matrices relevant for environmental risk assessment according to European Union regulations.

## Materials and Methods

**FIGURE 1:**
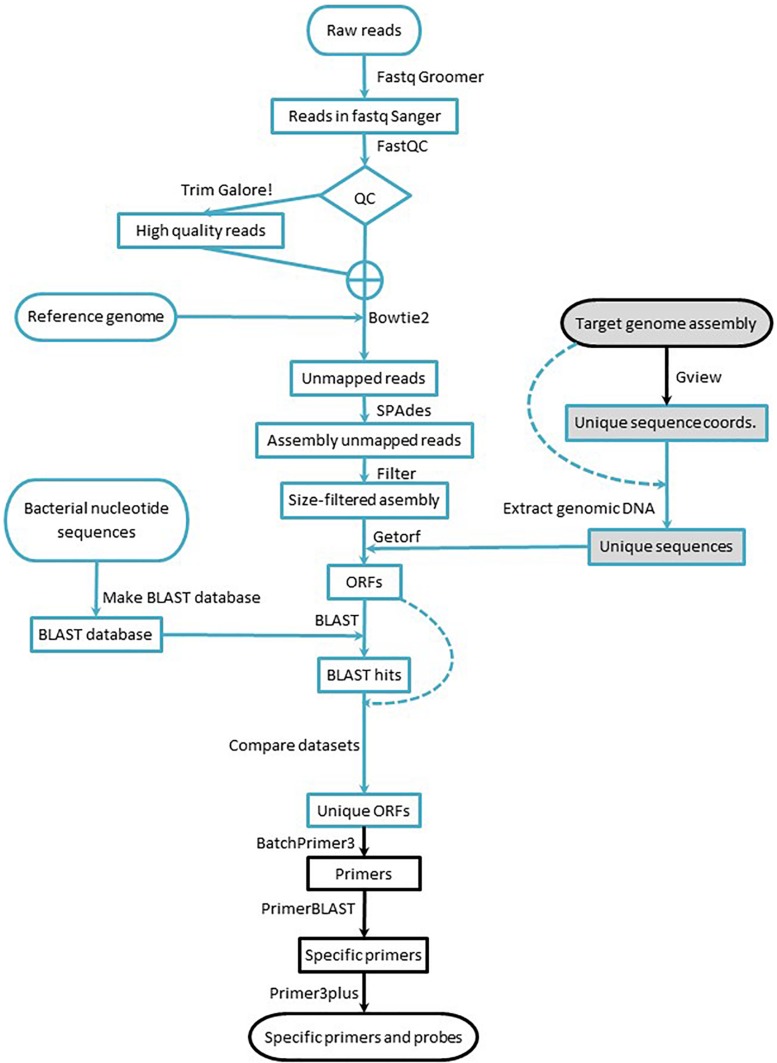
Workflow for marker design. Blue lines indicate processes run in the Galaxy environment, and black lines indicate processes run using online tools. Arrows indicate the workflow direction, and dashed lines indicate processes that require a dataset for a second time. Shaded elements denote the part of the workflow applied to data acquired with long read sequencing technology.

The workflow consists of: (i) identifying the strain-specific sequences, (ii) designing specific primer/probe sets for qPCR assays, and (iii) empirically verifying the performance of the assays, including their specificity toward the intended target strain. The first two stages (Figure 1) consist exclusively of computer work, but they are intended to be implemented by researchers with little or no bioinformatic background: Only a knowledge of the BLAST suite tools and work with spreadsheets are required; a familiarity with the Galaxy environment and NGS concepts are strongly advised. The workflow can be implemented starting with data from short or long read sequencing platforms and can be fully implemented using publicly available resources and a regular desktop computer (no matter the operating system) connected to the Internet.

### Genome Sequencing

The WGS (whole genome shotgun) sequences of three bacterial strains – namely B2017, B25, and B2021 – were obtained as described previously (Hernández and Fernàndez, 2017) using an Illumina NextSeq500 platform on a Nextera library through a NGS service provider (Genomix4life, Baronisi, Italy) and were assembled using the reference-assisted tool Reconstructor (Tranchida-Lombardo et al., 2018). The genomes of two additional bacterial strains, B24 and B410, were sequenced with a single SMRT cell each on a SMRTBell 10 kb library with a PacBio RSII platform and were assembled using the HGAP pipeline (RS_HGAP Assembly.3) (Chin et al., 2013). The genome sequences are available at the DDBJ/ENA/GenBank under the accession numbers CP043732 (B24), MTAY00000000 (B25), VRYF00000000 (B410), QWEF00000000 (B2017), and SMOF00000000 (B2021), and the raw reads, at the SRA at their respective Bioprojects.

### Pre-processing Raw Reads

Raw reads obtained from the short read sequencing platform were transformed to Fastq Sanger using Fastq Groomer (Blankenberg et al., 2010) to standardize quality scores. Then, the reads were quality-trimmed with TrimGalore!.^1^ To evaluate the impact of the quality trimming, the reads were submitted to FastQC^2^ before and after trimming. After visual inspection of the FastQC outputs, the set of reads – raw or trimmed – that was submitted for subsequent analyses was decided (Figure 1). All the aforementioned bioinformatic tools were implemented through the Galaxy Australia public server at https://usegalaxy.org.au (Afgan et al., 2016), and detailed description of all settings are provided in the Supplementary Material.

### Obtaining Strain-Specific ORFs

To obtain strain-specific ORFs from strains sequenced with short reads (B25, B2017, and B2021), the high quality reads were mapped to the corresponding reference genome [*Lysobacter enzymogenes* ATCC 29487Z78 (FNOG01) for B25, *Pseudomonas putida* KT2440 (NC_002947.4) for B2017, and *Pseudomonas fluorescens* SW25 (NC_012660.1) for B2021] with Bowtie2 (Langmead et al., 2009; Langmead and Salzberg, 2012). The paired reads that did not map to the reference genome were assembled *de novo* using SPAdes (Bankevich et al., 2012), and the resulting contigs with coverage over 10× were filtered by length (200 nt) (Blankenberg et al., 2010). Next, the ORFs were extracted from the contigs using EMBOSS getorf (Rice et al., 2000). The extracted ORFs were submitted to BLAST homology search against all bacterial nucleotide sequences in the GenBank (Altschul et al., 1990; Camacho et al., 2009; Cock et al., 2015). The BLAST results were cross-compared with the strain-specific ORFs to obtain ORFs with no significant BLAST hit. As in the previous section, all the bioinformatic tools were implemented through the Galaxy Australia public server at https://usegalaxy.org.au (Afgan et al., 2016), and details of the settings are provided in the Supplementary Material.

For genomes sequenced and assembled from long reads (B410 and B24), the strain-specific sequences were obtained comparing the target genome with a reference genome using the unique genome analysis tool in the GView Server (Stothard et al., 2017). The output of the unique genome analysis – a gff file with the coordinates of unique sequence stretches – was used to extract the unique sequences from the assembled genome with the GetFastaBed tool from the BEDTools suite (Quinlan and Hall, 2010). Next, the ORFs were extracted from the unique sequences as described for genomes sequenced with short reads technology. All bioinformatic tools were implemented through the Galaxy Australia public server at https://usegalaxy.org.au (Afgan et al., 2016) except for the unique genome analysis, which was carried out online via the GView Server (Stothard et al., 2017). After obtaining strain-specific ORFs, primer and probe design was carried out as described next.

### Primer and Probe Design

Strain-specific ORFs were submitted to BatchPrimer3 (You et al., 2008), setting the parameters to be compatible with qPCR with hydrolysis probe detection (see Supplementary Material). From all primer pairs, candidates were selected based on individual primer self-complementarity and primer pair complementarity. The candidate primer pairs and the amplicon sequence were submitted to PrimerBLAST (Ye et al., 2012) to verify that they showed no off-target among bacterial sequences deposited in the GenBank (Rice et al., 2000; Blankenberg et al., 2007; Ye et al., 2012) (see Supplementary Material for settings).

Finally, internal oligos were designed using Primer3Plus (Untergasser et al., 2007) for selected amplicons, setting oligo properties to serve as hydrolysis probe in qPCR assays (see Supplementary Material for settings and Supplementary Table 1 for the selected primer/probe sets). Primer pairs for which internal oligos could not be designed were discarded.

### Wetlab Verification of Primer/Probe Functionality and Specificity

The selected primer pairs were tested empirically for their functionality with their target strain by real-time PCR with intercalating dye chemistry and melting curve analysis (Light Cycler 480 II; Roche Life Science). For this, nutritive agar (NA) plates were streaked with the target strain. A single colony was picked, dipped in 50 μL sterile nanopure water and boiled for 10 min at 98°C. Serial dilutions of the boiled colony were used as template for real-time PCR reactions. Each reaction consisted of 5 μL SYBR Green I Master Mix (Roche Life Sciences), 0.5 μL each primer at 10 μM (Sigma), 2.5 μL template (serial dilutions of boiled colony), and 1.5 μL water. The thermal cycling conditions consisted of initial denaturing (10 min at 95°C), amplification [45 × (10 s at 95°C, 10 s at 60°C, and 15 s at 72°C)] and a melting curve (5 s at 95°C, 60 s at 65°C, and increasing temperature from 65 to 97°C at 0.11°C⋅s^–1^ with 5 fluorescence acquisitions⋅s^–1^; Light Cycler 480 II, Roche Life Science). The PCR efficiency (*E*) was calculated, amplifying serial dilutions of a template as described elsewhere (Bustin et al., 2009).

The primer pairs yielding an amplicon (as deduced form the Ct and the melting curve analysis) were tested for their specificity toward the intended target strain. For this, closely related bacterial isolates from the Futureco Bioscience in-house collection, classified by 16S sequencing (amplified with 8f and 1492r primers) with the primer 8f as described elsewhere (Eden et al., 1991; Jiang et al., 2006), were used as off-target strains (Supplementary Table 2). Although it is well described that this gene has little discrimination power within some genera, it is widely accepted as an approximation in bacterial taxonomy (Hayashi Sant’Anna et al., 2019). The template preparation, reaction composition and PCR conditions were as described before for testing primer functionality. Only primer pairs with no amplification at all, or amplifications with Ct > 35 and a melting curve clearly different from the intended target, were considered specific for the target strain.

### Setting Up Hydrolysis Probe-Based Triplex Assays

From the primer pairs with proven specificity toward the intended target strain, three were selected, and the previously designed hydrolysis probes – internal hybridization oligos labeled with a fluorophore each at their 5′ end and an adequate quencher at their 3′ – were purchased from Isogen (FAM- and CY5-labeled probes) or Jena Bioscience (R610-labeled probes) (Supplementary Table 1).

Using serial dilutions of target strain boiled colonies as template, hydrolysis probe-based real-time PCR reactions were set sequentially in monocolor, dual color, and triplex assays in order to flag possible primer/probe cross-inhibition that may impair PCR. The real-time PCRs with hydrolysis probe chemistry were performed on 20 μL including 10 μL PerfeCTa qPCR ToughMix (Quantabio), 1 μL each target mix (including the two primers at 10 μM each and the corresponding hydrolysis probe at 4 μM), and 7 μL template (boiled colony); the thermal cycling conditions were 3 min at 95°C (initial denaturing) and 45 × (15 s at 95°C and 60 s at 60°C), with fluorescence detection at the end of the extension step.

### Determination of the Absolute Limit of Detection (LOD)

For each target strain, the three selected amplicons were amplified individually by conventional PCR using Biotaq polymerase (Bioline) following manufacturer’s instructions in 50 μL-reactions using 4 μL of target strain boiled colony as the template. After purification (REAL Clean Spin PCR, Durviz) and verification of the amplification by agarose gel electrophoresis, the concentration of the amplicons was determined using the dsDNA Broad Range Assay (Denovix) in a microvolume fluorometer (DS-11 FX, Denovix). From the amplicon sequence and its mass concentration, the molar concentration was determined. Equimolar mixtures of the three amplicons, were used as absolute quantification standards. These standards were stored no longer than 5 days at −20°C, as recommended by Dhanasekaran et al. (2010).

Serial dilutions of the absolute quantification standards were amplified, in technical triplicates, using the triplex assay as described in the previous section. Ten more technical replicates of the most diluted triplicates that scored positive for amplification were repeated in order to define the LOD [i.e., the most diluted template amount at which > 95% of the technical replicates score positive (Bustin et al., 2009)].

For the determination of the LODs in pure cultures, single colonies of target strains were diluted in 100 μL H_2_O and mass-grown in an NA plate for 72 h at 26°C. The bacterial growth was collected in 1 mL sterile distilled water and the CFUs⋅mL^–1^ were determined by dilution plate counting. Serial dilutions of the culture were prepared with sterile distilled water ranging 10^9^–10^0^ CFU⋅mL^–1^. A 0.1-mL aliquot of each dilution was boiled for 10 min at 98°C and immediately frozen at −20°C until analysis. The LOD was determined using the boiled serial dilutions by the triplex real-time PCR assay using 7 μL of boiled bacterial culture as the template. The LOD values were calculated as described before for pure amplicons.

### Target Strain Detection in Water Samples

Freshwater samples were collected from the Anoia river at Sant Sadurní d’Anoia (herein “Anoia”; 41.429735, 1.799622; Barcelona, Spain), the Foix river at Santa Margarida i els Monjos (herein “Foix”; 41.321038, 1.661719; Barcelona, Spain), a well at Sant Cugat Sesgarrigues (herein “St. Cugat”; 41.363751, 1.753023; Barcelona, Spain), and the tap at the Futureco Bioscience facilities (herein “tap”; 41.355263, 1.732914; Barcelona, Spain) (see Supplementary Table 3 for details about water properties). Fourteen-mL water samples were inoculated with 1 mL liquid cultures of the different bacterial strains to a final concentration of 5⋅10^8^ CFU⋅mL^–1^ and stored under surface water-simulating conditions (inside a greenhouse with natural illumination and environmental temperature, from March 2018 to February 2019) and groundwater-simulating conditions (in the dark at 4–6°C). The standard calibration curves were prepared using serial dilutions of target strains on the liquid matrix (i.e., water from different sources). After mixing thoroughly, 0.1-mL aliquots were boiled as described before for template preparation and analyzed by triplex qPCR as described before.

### Target Strain Detection in Soil Samples

The greenhouse substrate, used for in-house efficacy trials with B25 in potato plants, was made of peat and perlite (3:1; v:v); the field substrates were taken from field efficacy trials in Vilanova d’Arousa (42.5422353, −8.750556669; Pontevedra, Spain) and Picanya (39.4226006, −0.43878345; Valencia, Spain), with B2017 and B2021, respectively, on potato plants, too. The two field substrates were from plots under regular tillage and with good draining, but the former was a loam, and the later was a sandy loam.

In the aforementioned efficacy trials, addressed to soil pathogens, aqueous suspensions of the mBCAs were applied directly to the soil (spray to seed potatoes in the furrow, or drench irrigation in greenhouse trials). DNA was extracted form soil samples using the Quick-DNA Fecal/Soil Miniprep kit (Zymo Research), following manufacturer’s instructions, for a final elution volume of 50 μL. The resulting DNA was PCR-amplified using the triplex real-time qPCR protocol described before. For each soil type analyzed, a standard calibration curve was built. For this, water-saturated soil samples were added serial dilutions of the target bacteria, mixed thoroughly, and let to stand for 2 h at room temperature. Then, DNA was extracted as described before from 0.2-mg aliquots, and the calibration curves were built from the Ct values and the strain concentration.

### Target Strain Detection in Plant Samples

Potato plants were removed from the soil and shaken to remove as much substrate as possible. Then tissue samples were harvested, snap-frozen in dry ice, and stored at −80°C until analysis. Frozen plant tissue samples were grinded in a ball mill, and DNA was extracted from them with the REAL Pure Spin Plants and Fungi DNA kit (Durviz) following manufacturer’s instructions, for a final elution volume of 50 μL. The resulting DNA was then PCR-amplified using the triplex real-time qPCR protocol described before. For each plant sample type analyzed (potato roots and shoots), a standard calibration curve was built by adding serial dilutions of the target strain to 0.2-mg aliquots of frozen plant samples and processing the mixtures as described for the samples. The calibration curves were built from the Ct values and the strain spike concentrations.

## Results

### Workflow Output

The number of input reads for genomes sequenced with short read technology varied among strains, from 1,797,786 to 4,507,278 (Table 1). After quality trimming, 12.4–14.8% of the reads were discarded (Table 1). The percentage of trimmed reads that mapped to the reference genome varied from 31.2 to 78.6% (Table 1). The unmapped reads were assembled in a number of smaller contigs (142–617). On the other hand, the strains sequenced with long read technologies were assembled in one (B24) and nine (B410) contigs (Table 1). The number of potentially new ORFs produced with the workflow ranged from 751 to 13,929, with average lengths between 345 and 486 nucleotides (Table 1), and the hard disk space required for the workflow was about 30 Gb for strains sequenced with short reads and about 4 Gb for those sequenced with long reads (Table 1). The number of primer pairs obtained ranged between 11,074 (B24) and 697 (B2021).

**TABLE 1:** Workflow performance on the different strains.

Parameter	Long reads		Short reads		
	B24	B410	B25	B2017	B2021
Raw reads (×2)	N/A	N/A	4,507,278	1,797,786	4,402,926
Trimmed reads (×2)	N/A	N/A	3,839,370	1.590,622	3,857,039
Unmapped reads (unmapped reads %)	N/A	N/A	2,618,677 (68.2%)	49,7303 (31.3%)	303,1250 (78.6%)
New contigs*	1	9	479	617	142
**New potential ORFs**					
Number	11,074	3,986	13,929	4,338	751
Avg. length (nt)	396	352	486	345	370
Memory (Gb)	4.16	4.09	29.01	26.65	33.26
Primers pairs produced	11,074	3,986	7,767	3,911	697

*For B24 and B410, “new contigs” refers to the *de novo* genome assembly.

### Performance of Obtained Markers

From all the primer pairs obtained, only a small proportion was tested empirically (between 10 and 30, depending on the strain) for specificity, and only a proportion of them (18–67%) resulted specifically toward the intended target (data not shown).

When used in qPCR assays with intercalating dye, the primers selected for multiplex hydrolysis probe qPCR assays showed *E* values of 2.00 on average, although some of them showed poor performance (Supplementary Table 4). Overall, shifting from intercalating dye to hydrolysis probe chemistry did not alter the *E* values (1.96 on average) in the different assays, although some particular cases show significant changes (e.g., the channel R610 in B24, the R610 channel in B2017, and the CY5 channel in B2021; Supplementary Table 4). Increasing the degree of multiplexing, overall, maintains the *E* of each single primer/probe set so the triplex assays maintain an *E* close to 2.00 (1.98 on average) although, again, some particular cases show poor performance (e.g., the R610 and CY5 channels in B410, the FAM channel in B2017, the FAM channel in B2021; Supplementary Table 4). From the primer pairs that were verified empirically to yield an amplicon, all primer/probe sets (Supplementary Table 1) were specific for the intended target – as compared to the off-targets shown in Supplementary Table 2 – regardless of the qPCR chemistry and multiplexing degree used for the analysis.

When the triplex assays were run using pure amplicon as template, the limits of detection ranged between 1 (B25) and 122 (B410) copies⋅μL^–1^ and the linearity, between 5 (B2021 and B2017) and 7 (B24, B25, and B410) orders of magnitude (Supplementary Table 5).

### Monitoring mBCAs in Environmental Samples

**FIGURE 2:**
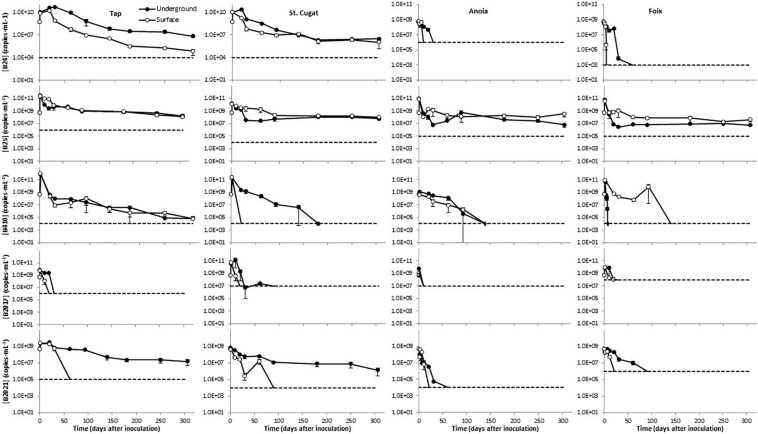
Persistence of five microbial biocontrol bacterial strains in waters of different natures, stored under conditions simulating surface and groundwater. Dashed lines represent the limit of detection; data points below detection limits are omitted. Negative controls (untreated water) yielded concentrations below detection limit in all cases (not shown).

Overall, after an initial growth that lasted for 1–30 days, depending on the water source and storage conditions, the levels of B24 declined with the time in the different freshwaters tested (Figure 2). This decay was more pronounced in surface-simulating conditions, particularly in tap water, as compared to groundwater-simulating storage. B24 was particularly sensitive in Anoia and Foix river waters, where it dropped below detection limits (10^6^ and 10^3^ copies⋅mL^–1^, respectively) by day 50 (Figure 2). B25 showed an initial growth during the first day and, overall, the declines observed in this mBCA were less evident than those in B24. After day 50, such decline was only appreciable in tap water (Figure 2). In the water samples of St. Cugat, Anoia, and Foix, there was an initial decrease during the first 50–100 days, but the B25 levels remained constant thereafter (Figure 2). In addition, there is little difference in the B25 levels in freshwaters when stored under surface- or groundwater-simulating conditions (Figure 2). B410 showed the same initial growth immediately after inoculation and declined thereafter (Figure 2). In tap water, the decline was slow and steady to reach concentrations below 10^5^ CFU⋅mL^–1^ by the end of the experiment (304 days; Figure 2). In tap water, B410 showed the same dynamics under surface- and groundwater -simulating conditions (Figure 2). In the St. Cugat well water, this mBCA dropped below detection limits (10^4^ copies⋅mL^–1^) by day 20 in surface-simulating conditions and by day 180 in groundwater-simulating conditions (Figure 2). In Anoia river water, B410 levels dropped below the detection limit (10^4^ copies⋅mL^–1^) by day 140, regardless of the storage conditions (Figure 2). In Foix river water, the B410 levels dropped below detection limits (10^4^ copies⋅mL^–1^) by day 20 in groundwater-simulating conditions and by day 140 in surface-simulating conditions (Figure 2). The levels of B2017 fell below the detection limits in all water types before day 40 (in St. Cugat well water B2017 levels were in the edge of the detection limit of 10^7^ copies⋅mL^–1^ from day 30 to day 90 (Figure 2). However, it has to be noted that the detection limits for this mBCA are significantly higher than for the other mBCAs analyzed (Figure 2). Finally, B2021 stored under surface-simulating conditions dropped below detection limits within 100 days in all water types (Figure 2). In tap and St. Cugat well waters stored under groundwater-simulating conditions, B2021 levels declined steadily throughout the entire experimental period, reaching values about 1.5 and 2.5 orders of magnitude lower than the initial inoculum by day 304, respectively (Figure 2). This mBCA declined much faster in Anoia and Foix river waters stored in groundwater-simulating conditions, dropping below detection limits (10^4^ and 10^6^ copies⋅mL^–1^; respectively) by day 60 and 90, respectively (Figure 2). For space reasons, the mBCA concentrations shown in Figure 2 correspond only to the FAM-labeled marker, but the results with the R610- and CY5-labeled makers do not differ significantly from the FAM channel.

**FIGURE 3:**
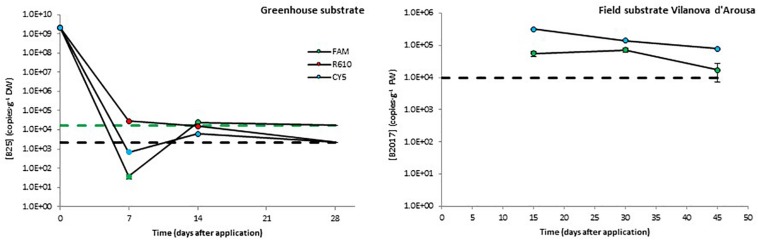
Persistence of two biocontrol bacterial strains (B25 and B2017) in soils of different natures. The presence of B2021 in the substrate from a field trial in Picanya was below detection limits (2.4 × 10^3^ CFU⋅g^–1^) 36, 64, and 86 days after application. Bacterial concentrations were measured with three markers per strain (only two for B2017), labeled with different fluorophores (FAM, CY5, and R610 [LightCycler Red 610]). Dashed lines represent the limits of detection (in black when those of several channels overlap). Data points below detection limits are omitted except for those helpful to obtain a view of the overall trend, which are shown with no edge. Negative controls (untreated soils) yielded concentrations below detection limit in all cases (not shown).

After inoculation in greenhouse substrate, the levels of B25 decreased rapidly during the first 7 days. This decrease was about five orders of magnitude in the FAM channel and over six orders of magnitude in the R610 and CY5 channels, the latter two showing values below the detection limit. From day 7 to day 15 after inoculation, the levels of B25 remained constant or increased slightly as to turn detectable in the R610 and CY5 channels. Finally, by the end of the experiment, the levels of B25 dropped below detection limit in all three channels (Figure 3).

Taking into account the plantation density, the concentration of the mBCAs in the application solution, and the volume applied, B2017 and B2021 were applied at 1.5 × 10^7^ and 7.8 × 10^8^ CFU⋅plant^–1^ rates, respectively; these dosages were defined based on previous, unpublished field efficacy trials. However, the initial dosage in field experiments cannot be defined in terms of CFU⋅g^–1^ soil since these are open systems. The levels of B2017 in the substrate from the field trial in Vilanova d’Arousa were around 10^5^ CFU⋅g^–1^ (FAM channel) and 5⋅10^6^ CFU⋅g^–1^ (CY5 channel; Figure 3) 15 days after application. These levels decreased to reach 1.7 × 10^4^ and 8.0 × 10^4^ CFU⋅g^–1^, respectively, 45 days after inoculation (Figure 3). The standard calibration curve in the R610 channel showed poor regression coefficient, so this channel was discarded from the analyses. The presence of B2021 in the field substrate from Picanya was below the detection limit of 2.4⋅10^3^ copies⋅g^–1^ in all sampling points (7, 14, and 28 days after the inoculation).

**FIGURE 4:**
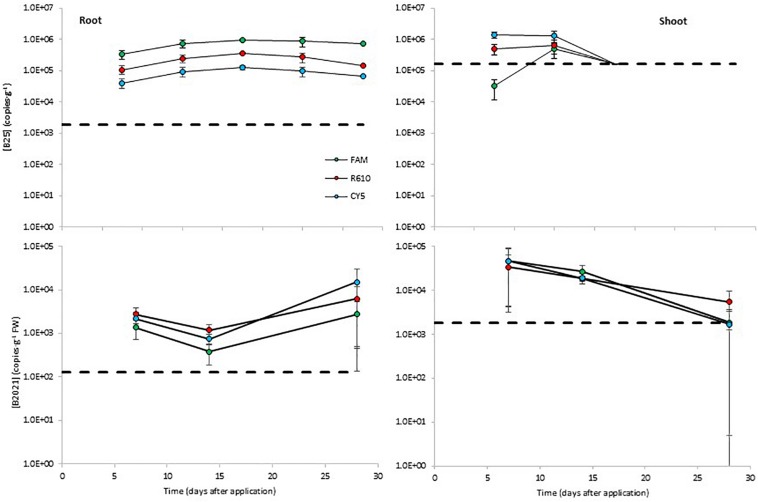
Colonization of potato plant tissues by the biocontrol bacterial strains B25 and B2021 (B2017 was not detected over the limit of detection of 2.6 × 10^5^ copies⋅g^–1^ FW). Bacterial concentrations were measured with three markers per strain, labeled with different fluorophores (FAM, CY5, and R610 [LightCycler Red 610]). The dashed lines represent the limits of detection; data points below detection limits are omitted. Negative controls (untreated plants) yielded concentrations below detection limit in all cases (not shown).

The levels of B25 in potato roots remained steady around 10^5^ copies⋅g^–1^. The B25 levels determined with the FAM channel were about 0.5 log higher than those determined with the R610 channel, and the levels measured with the CY5 channel were about 0.5 log lower than with the R610 channel (Figure 4). In aerial tissues, B25 remained steady around 10^5^–10^6^ copies⋅g^–1^ from day 7 to day 15 and declined thereafter below detection limits (Figure 4). B2021 showed similar dynamics as B25 in plant tissues. In root tissue the levels of B2021 remained stable from day 7 to day 28, around 10^3^–10^4^ copies⋅g^–1^; and in aerial tissue, declined from day 7 to day 28, from ca. 5** ×** 10^4^ to ca. 2** ×** 10^3^ copies⋅g^–1^, although in this case B2021 was effectively detected 28 days after inoculation (Figure 4). B2017 was not detected over the LOD in potato plant tissues.

## Discussion

### Performance of the Workflow

Applied to the strains shown hereby, the workflow yielded a large number of potential primer pairs from which a small proportion was tested empirically. This high number reflects a considerable amount of strain-specific ORFs and contigs identified, representing a very large proportion of the genome assembly drafts. Indeed, the percentage of unmapped reads (in the case of mBCAs sequenced with short reads) was between 31 and 79% (Table 1). This may be due to the phylogenetic distance of the genomes used as reference toward the test mBCAs. In the course of this research, closer reference genomes have been released, so using them would most likely reduce the number of unmapped reads and therefore the number of output primer pairs. The high number of potential markers obtained with the proposed workflow contrasts with the few – in the order of tens – markers obtained usually identified in RAPD or AFLP experiments (Hermosa et al., 2001; Dauch et al., 2003; Felici et al., 2008; Xiang et al., 2010; Perez et al., 2014). The workflow offers a number of tuning alternatives that would yield different, yet valid, output. For instance, the use of genomic DNA sequences instead of ORFs may yield a larger number of potential markers; in the present manuscript, ORFs were used in order to restrain the amount of strain-specific sequences and flag possible functional information about the markers. On the other hand, if a complete chromosome assembly is available for the target strain genome, several genomes may be used as reference using the GView unique genome analysis tool, and the amount of output markers would be restrained. Another alternative is to set primer and probe design parameters for higher astringency and different qPCR detection chemistries. In the cases presented here, multiplex hydrolysis probe detection was chosen for the possibility to include multiple markers within a single qPCR reaction; even more robust assays would be achieved using amplification panels followed by massive sequencing. Overall, the workflow presented hereby offers the possibility of tuning experimental settings in order to obtain the best balance among the number of false positive makers (e.g., unspecific or not functional primers) and the total amount of markers obtained. This tuned astringency may be also applied when selecting markers by other methods such as RAPD or AFLP (Williams et al., 1990; Vos et al., 1995) for sequence characterization; but due to the low amount of strain-specific bands usually obtained, this tuning is rarely applied. The time requirement to run the entire workflow depends on the Internet connection and the availability and workload of the servers hosting the tools used (Galaxy Australia, GView, BatchPrimer3, primer-BLAST, and Primer3Plus). The Galaxy Australia server was chosen because it has all the necessary tools installed but any other Galaxy server may be used instead. During the course of manuscript preparation, it was noticed that the Galaxy Europe server^3^ has also all of the necessary tools readily installed. The most time-consuming steps are downloading the bacterial nucleotide sequences from the GenBank and creating the BLAST database with them (Figure 1): These steps take several hours each. Thus, although the workflow can be completed in a working day, most often it takes two or three days. Starting from the raw reads, this workflow is considerably faster than identifying RAPD/SCAR markers and consumes much fewer fungible materials, while wetlab verification of marker performance is similar using the workflow presented here an RAPD or AFLP markers.

The workflow was initially intended to run with raw reads from short read sequencing platforms (namely Illumina), but markers for candidate strains sequenced with long read technologies (namely PacBio) may be obtained using the GView unique genome analysis tools (Petkau et al., 2010; Stothard et al., 2017) and the target mBCA genome assembly draft, as shown in Table 1 and Figure 1.

Real-time qPCR assays showed variable performance but, overall, the absolute detection limits using pure amplicon as template were close to a theoretical LOD (Supplementary Table 5; Bustin et al., 2009). As expected, when applied to different sample matrices the LODs increased significantly. For freshwater samples, the LODs ranged from 10^3^ to 10^8^ copies⋅mL^–1^ (Figure 2); for soil samples, from 10^3^ to 10^4^ copies⋅g^–1^ (Figure 3); and in potato plant tissues, from 10^2^ to 10^5^ copies⋅g^–1^ (Figure 4). The lowest values are in line with those reported by other authors using qPCR methods (Soto-Muñoz et al., 2014; Rotolo et al., 2016; Daranas et al., 2018). On the contrary, in some cases, it is evident that further optimization of the method should be carried out (e.g., B2017 in water samples; Figure 2). The variability in the detection limits is, in part, associated to the mBCA itself, but the sample matrix also exerts a strong influence on the LOD in some cases (e.g., Anoia water for B24). Thus, further qPCR optimization should be considered for particular sample matrices. In the present work, among several hydrolysis probe-compatible qPCR master mixes, the PerfeCTa qPCR ToughMix (Quantabio) gave the best results, particularly in soil samples (comparison data not shown). But even after choosing the best performing qPCR master mix the qPCR was not optimal in all cases, where the *E*s were too low (down to 1.834) or too high (up to 2.455) (Supplementary Table 4). Thus, the inclusion of a calibration standard curve for each matrix is mandatory for an absolute quantification. The results obtained with the different channels for each mBCA and sample matrix are in concordance with one another showing differences below 1 log in the mBCA concentration. Still, in some cases, the concentrations measured by the different channels differ significantly (e.g., B25 greenhouse substrate 7 days after inoculation). It was verified that the primer/probe sets did not show multiple targets within the target strain genome taking advantage of the assemblies available, but contamination of the DNA by RNA may be possible since ORFs were chosen as strain-specific sequences. Thus, treating the extracted DNA with RNase might solve this issue but further work is needed to elucidate the source of this variability. Nevertheless, all channels show the same trend in all matrices analyzed, which validates the multiplex qPCR assays. In addition, not all primer pairs yielded by the workflow show absolute specificity toward the intended target. This is unavoidable because the number of strains related to the intended target strain that have DNA sequences available in the GenBank is very limited compared to what it is expected in nature. On the other hand, the more strains included in experimental validation of specificity, the better; but the number of strains tested must be kept within manageable numbers. Overall, considering that the qPCR assay includes the analysis of three markers simultaneously and that primers tested are specific to the intended target strain as compared to the GenBank and 6–47 additional closely related strains (Supplementary Table 2), it is safe to assume that the method is specific for the target strain. Furthermore, in case false positives are suspected, additional primer/probe sets could be easily tested.

### Dynamics of the mBCAs in Environmental Samples

The data shown in Figure 2 provide evidence that the different strains show differential sensitivity to the nature of the water source and the storage conditions. Overall, Anoia and Foix river waters, which show highest turbidity (Supplementary Table 3), were the least favorable to the tested strains; B25 is the only mBCA strain, among those tested, that reaches a stable population after the experimental period; and only B2021 was consistently sensitive to a particular storage condition – surface water-simulating conditions – in all water sources (Figure 2). All strains showed concentration decreases over 2 logs (i.e., an over 99% decrease) throughout the experiment, except B25, which showed levels at the end of the experimental period similar to those at the beginning or decreases less than 2 logs (i.e., a less than 99% decrease; Figure 2). Soil is a particularly challenging matrix for DNA detection due to its physical properties and the difficulty to avoid deleterious contaminants in purified DNA (Rajendhran and Gunasekaran, 2008). With the method presented here, mBCAs can be effectively monitored in this matrix, as shown in Figure 3. For mBCA monitoring assays in open systems, it is recommended to take samples immediately after application to have the initial mBCA levels on a substrate weight basis. Alternatively, assumptions on soil depth percolation based on application method (foliar spray, drip irrigation, inundation, etc.), tillage, crop interception, soil properties, etc., would have to be applied to obtain an initial mBCA concentration in a soil weight basis.

## Conclusion

An amenable workflow for the design and verification of qPCR markers for the detection of bacterial strains, is presented herein. This workflow starts from genome sequencing data – alternatively from genome assemblies – and produces a high number of marker sequences for the design of qPCR assays, minimizing the wetlab workload and using bioinformatic tools accessible to most researchers. The application of these markers is flexible to suit researchers’ needs. As an example, the work presented herein shows the application of the markers produced to track biocontrol strains in water, soil, and plant tissue samples in hydrolysis probe-based triplex qPCR assays. However, nothing precludes researches from using the markers obtained with the presented workflow in other ways such as vPCR, *in situ* hybridization, most probable number-PCR (MPN-PCR), etc., or in other sample matrices (e.g., air, blood, or stool) depending on the specific objective of the study.

## Data Availability Statement

The datasets generated for this study can be found in the GenBank MTAY00000000, GenBank VRYF00000000, GenBank QWEF00000000, and GenBank SMOF00000000.

## Author Contributions

IH conceived the work, performed computer and wetlab work, and wrote the manuscript. CS performed computer and wetlab work. RM and CS performed the wetlab work. CF supervised the work and revised the manuscript.

## Conflict of Interest

The use of B24, B25, B410, B2017, and B2021 as biocontrol agents is subjected to a patent application each. IH, CS, RM, and CF were employed by Futureco Bioscience S.A.

## Footnotes

1 http://www.bioinformatics.babraham.ac.uk/projects/trim_galore/

2 http://www.bioinformatics.babraham.ac.uk/projects/fastqc/

3 https://usegalaxy.eu/
