# Supplementary material for: Design of Bacterial Strain-Specific qPCR Assays Using NGS Data and Publicly Available Resources and Its Application to Track Biocontrol Strains
Source: Front Microbiol. 2020 Mar 10;11:208. doi: 10.3389/fmicb.2020.00208 (PMC7077341; doi:10.3389/fmicb.2020.00208)
Supplement: Supplementary file 1 [file Data_Sheet_1.PDF]

Detailed procedure for the identification of strain specific primer/probe sets.

The workflow is summarized in Figure 1 of the main text and the empirical verification of the functionality and specificity of the primer/probe sets is detailed in the main text.

Note that the results from different tools, the parameters required and the appearance of some tools may vary slightly from those shown hereby due to upgrades. Still, it is advised to use the latest versions available so long that the changes do not modify the workflow essence and, if necessary, consult the bibliography to understand the changes observed.

To undertake the workflow, one needs to have available the raw reads from a whole genome sequencing experiment.

0. Upload the data to Galaxy Australia.

Background knowledge of the Galaxy environment is required to proceed with the workflow so those new to it should get familiar on how to navigate through the different panels, how to up- and down-load or visualize data, etc. For this, please, explore the Galaxy help resources.

0.1. Download the reference genome.

Go to the PubMed web site (https://www.ncbi.nlm.nih.gov/pubmed), check the section "Genome" from the drop-down menu and type the species in the search box. Follow the link to the available genomes.

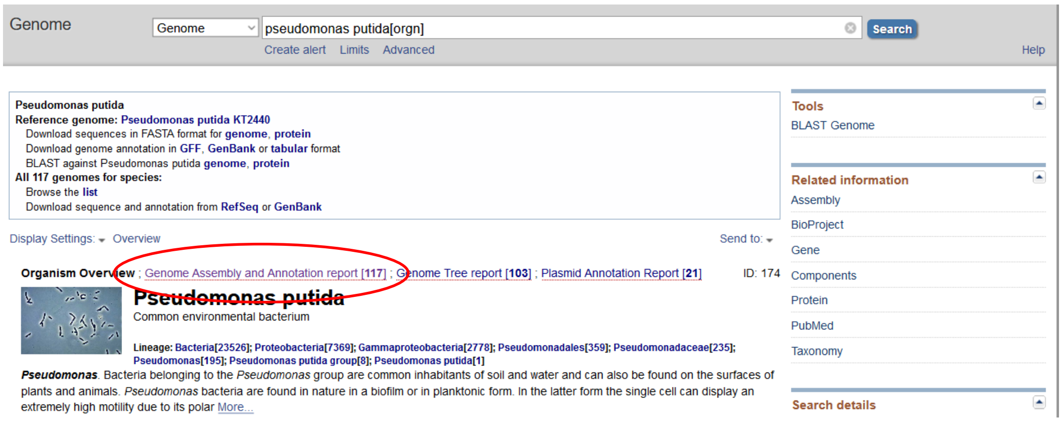

From the list of the available genomes, follow the link to the FTP of the strain of interest (Refseq or GenBank) and download the file with the nucleotide sequence in fasta (the file named *_genomic.fna.gz).

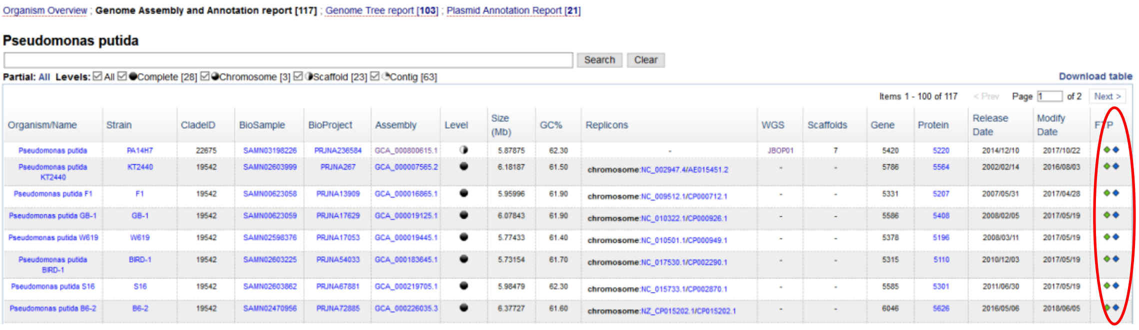

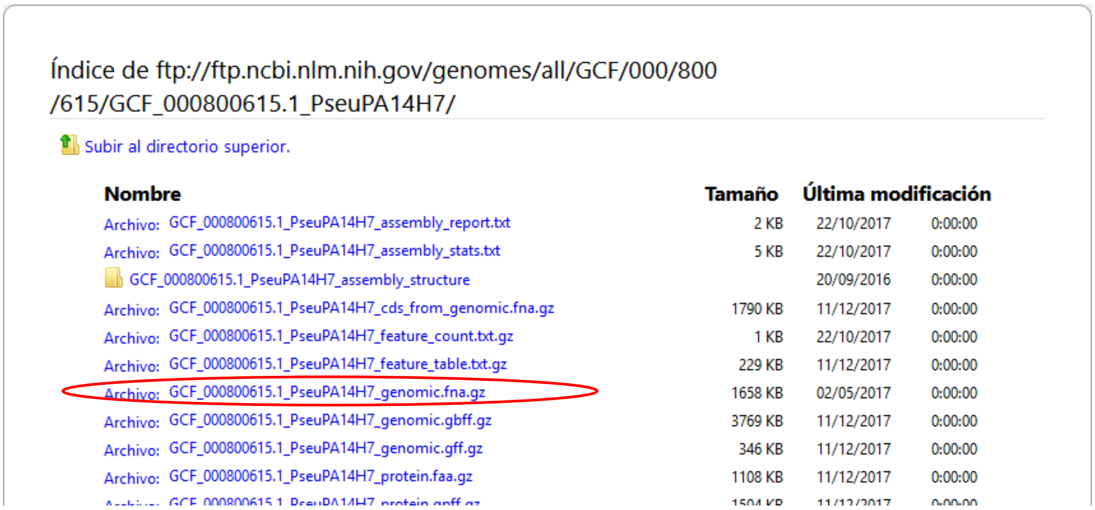

0.2. Download the bacterial nucleotide database.

Go to the PubMed web site (https://www.ncbi.nlm.nih.gov/pubmed), search for “bacteria” in the taxonomy database and follow the links to “Bacteria (eubacteria)” in the taxonomy browser.

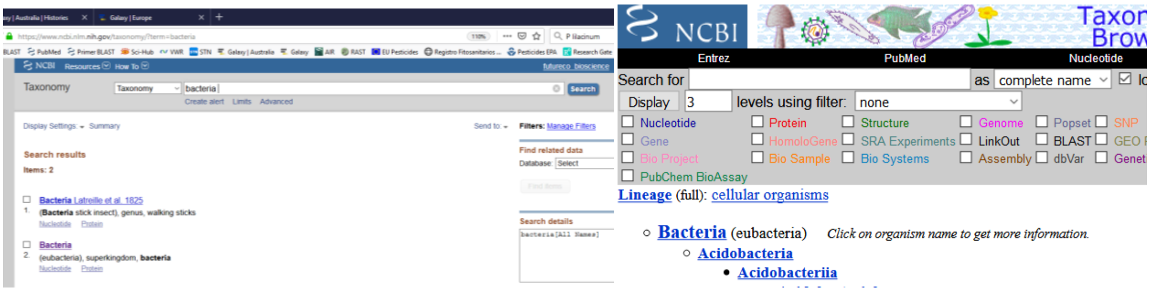

Click on the Nucleotide subtree links to produce all bacterial nucleotide sequences deposited so far.

Entrez records		
Database name	Subtree links	Direct links
Nucleotide	58,791,084	24
Protein	676,688,825	29,199
Structure	54,211	-
Genome	25,291	-
Popset	62,868	12,208
Conserved Domains	19,420	8,683
GEO Datasets	87,158	2,047
PubMed Central	786,260	-

Download the sequences in fasta format using the “send to” tool. This step takes a few hours due to the size of the file (>50 Gb by September 2019).

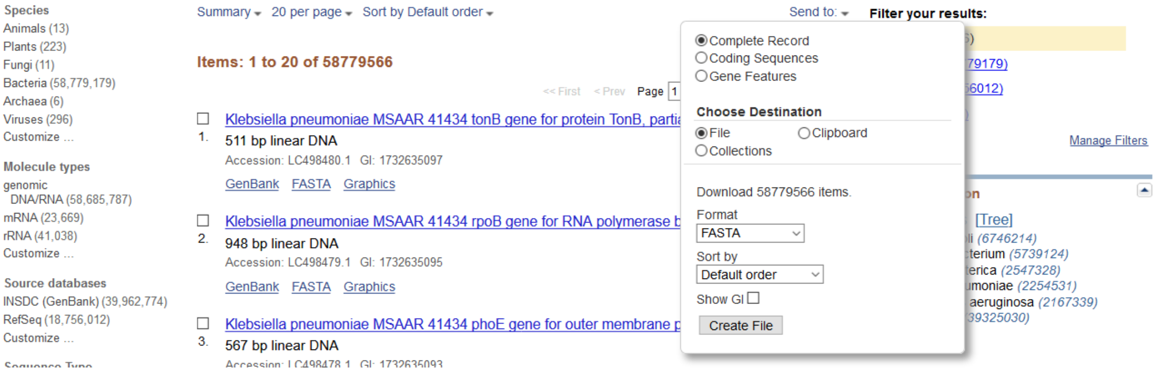

0.3. Upload the files to Galaxy Australia.

Go to the Galaxy Australia web site (https://usegalaxy.org.au/), register if not registered yet, log in and create a new history.

Upload the files with the raw reads obtained in the sequencing experiment (fastq files), the reference genome (fasta) and the bacterial nucleotides to the FTP server of Galaxy Australia using the preferred FTP software (see https://usegalaxy-au.github.io/posts/2019/03/18/new-ftp-upload-url/plain.html for details). Paired-end libraries are the most common strategy in bacterial whole genome sequencing projects; if this type of libraries were sequenced, as in the case presented in the manuscript, make sure to upload the two files of each pair. Once transferred, upload the files to the history (Get Data>upload a file from your computer).

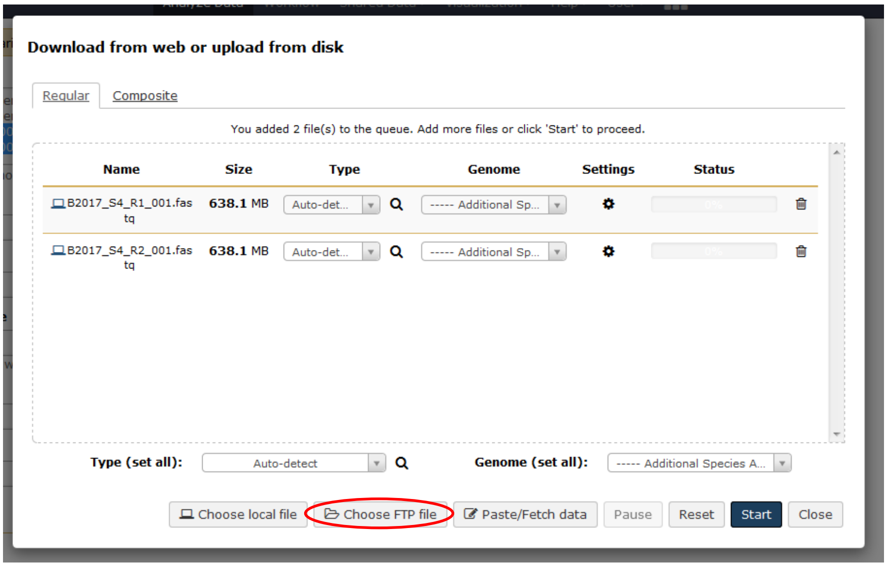

(The reference genome may be also upload directly from the user's computer (Get Data>upload a file from your computer)).

1. Pre-processing raw reads.

1.1. Transform fastq to fastq Sanger.

Transform the fastq files to fastq Sanger using FASTQ Groomer (3) (NGS: QC and manipulation>FASTQ Groomer) to standardize the quality scores so that all Galaxy tools can work with those files.

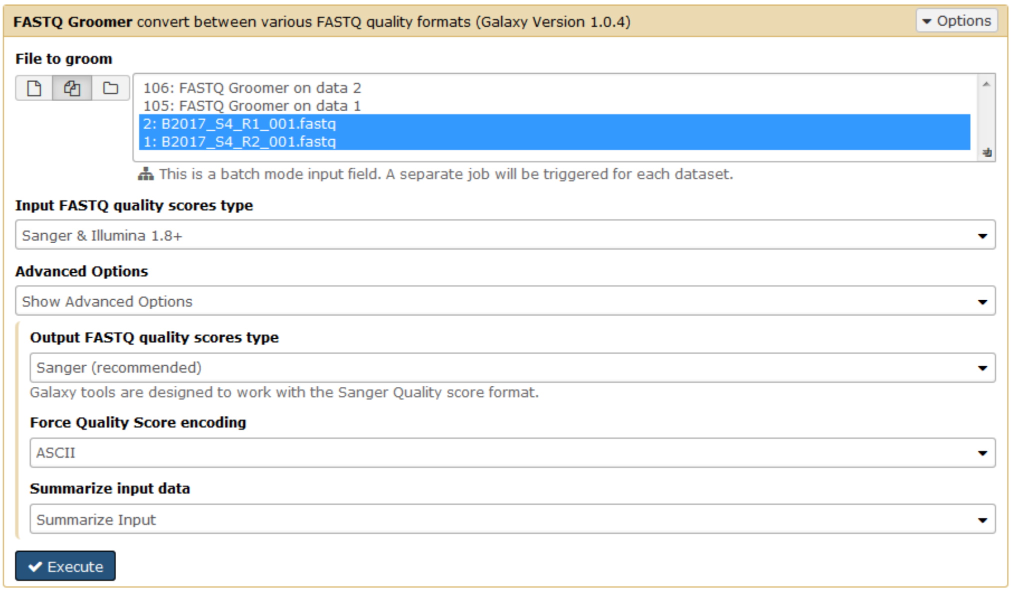

1.2. Quality control of the raw reads.

Run FastQC (4) (NGS: QC and manipulation>FastQC) to check the quality of the raw reads.

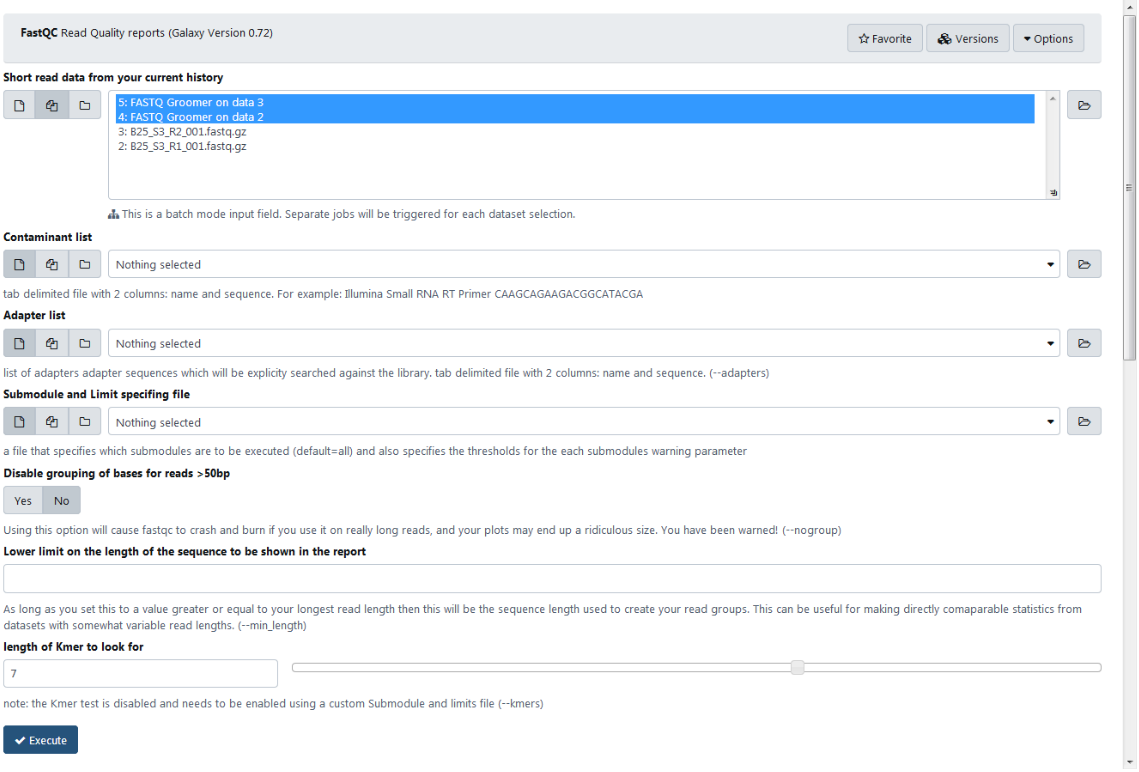

1.3. Trimming (optional).

Some service providers deliver trimmed data so this step is sometimes unnecessary. If the initial quality control of the raw reads (previous step) shows high quality data, this step and the next one can be omitted.

Trim the low quality sequences using Trim Galore! (5) (NGS: QC and manipulation>Trim Galore!) to discard low quality reads and portions of them, and reads that become too short after trimming. Set the Trim Galore! Parameters as follows:

- Is this library paired- or single-end? Paired-end.
    i. Reads in FASTQ format: reads pair 1.
    ii. Reads in FASTQ format: reads pair 2.
    iii. Adapeter sequence to be trimmed Automatic detection.
    iv. Trims 1 bp off every read from its 3' end: 1.
    v. Remove N bp from the 3' end of read 1: 1.

vi. Remove N bp from the 3' end of read 2: 1.

- Trim Galore! Advanced Settings: full parameter list.

  i. Trim low-quality ends from reads in addition to adapter removal: 30.

  ii. Overlap with adapter sequence required to trim a sequence: 1.

  iii. Maximum allowed error rate: 0.1.

  iv. Discard reads that became shorter than length N: 20.

  v. Generate report file: Yes.

     1. Specify... reads: do not output unpaired reads.

  vi. Leave the remaining parameters as set by default.

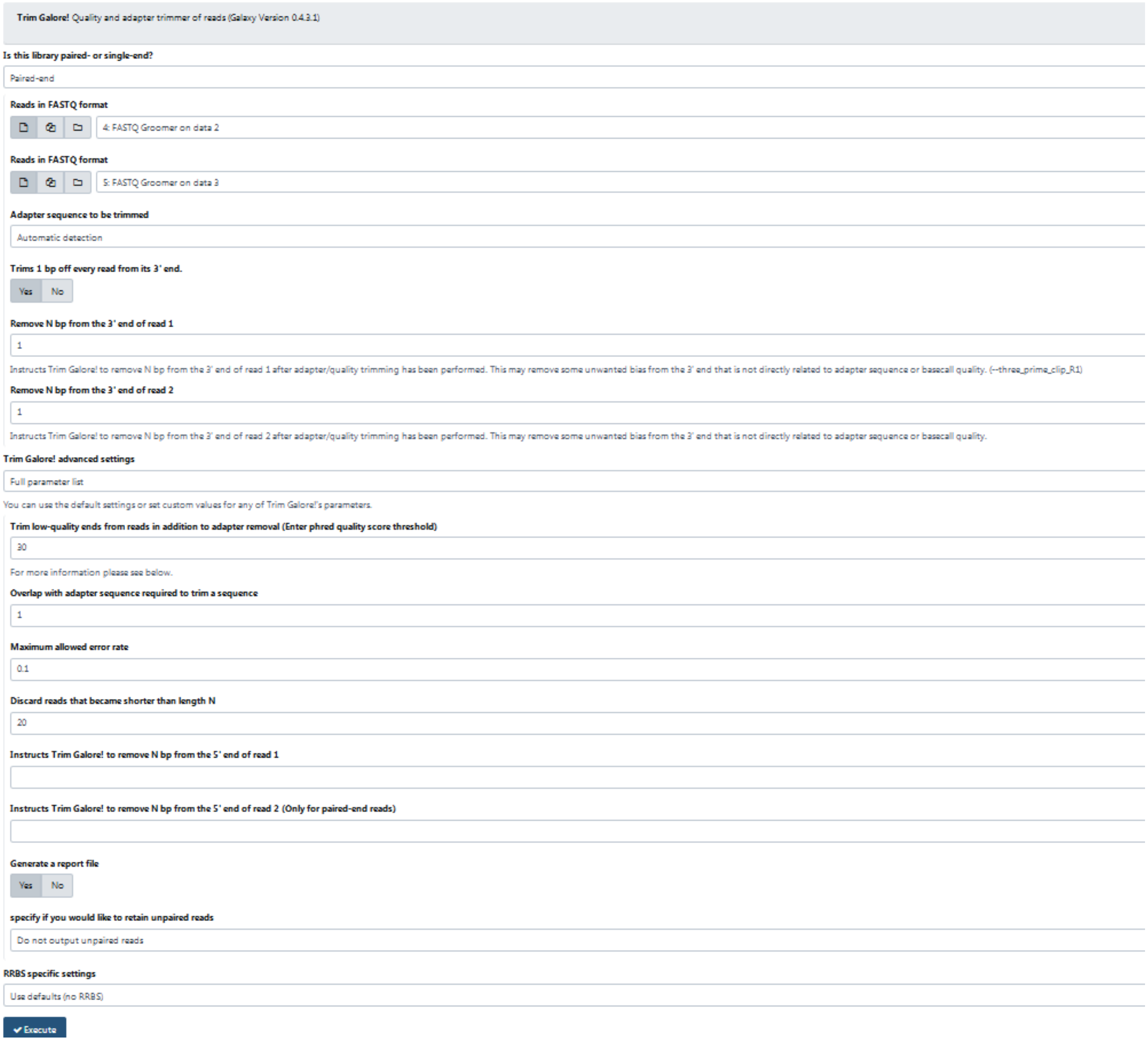

1.4. Quality control of the trimmed reads (optional).

If the trimming step has been performed, repeat the quality control (NGS: QC and manipulation>FastQC) to see if trimming improved the quality of reads (see http://www.bioinformatics.babraham.ac.uk/projects/fastqc/ to see how “good” and “bad” data look like). After trimming, one should note that:

- The Q values increase.
- The abundance of each base is constant through the reads (when analysing data from a Nextera library a distortion in the first 15 nucleotides is observed since the transposase cuts are not completely random).
- The GC content does not differ significantly from what was expected based on the reference genome.
- There are not overrepresented sequences (k-mers or adapters)

Before trimming After trimming

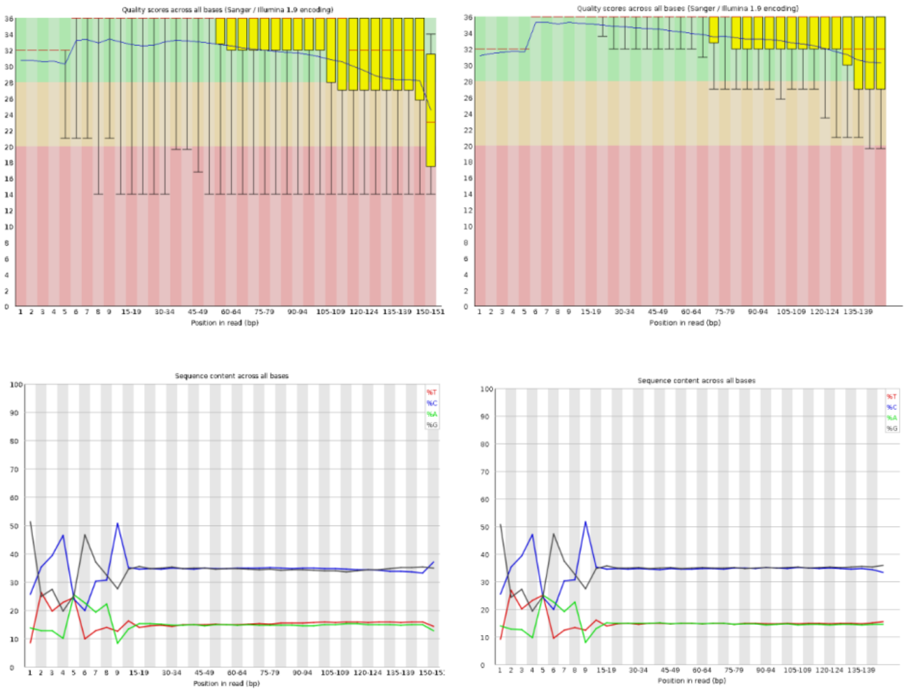

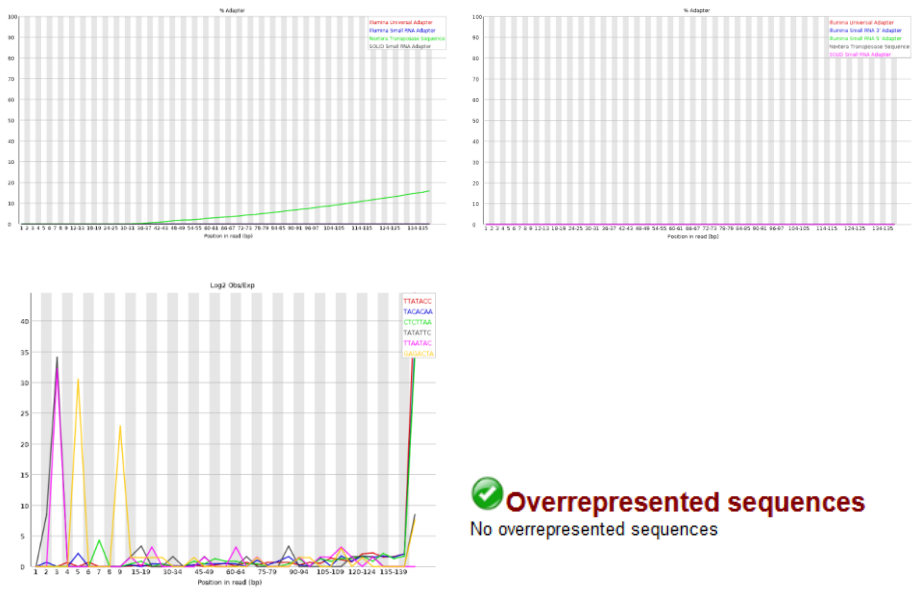

2. Obtaining strain-specific ORFs.

2.1. Map the reads to the reference genome.

Map the high quality reads to the reference genome using Bowtie2 (6) (NGS: Mapping>Bowtie2) to obtain the reads that do not align with the reference genome (unmapped reads). Set the Bowtie2 parameters as follows:

- Is this single or paired library: Paired-end.
- FASTA/Q file #1: Read pair 1.
- FASTA/Q file #2: Read pair 2.
- Write unaligned reads (in fastq format) to separate file(s): Yes.
- Write aligned reads (in fastq format) to separate file(s): No.
- Do you want to set paired-end options? No.
- Will you select a reference genome from your history or use a built-in index? Use a genome from the history and build index.
    - Select reference genome: Reference genome in fasta.

- Set read groups information? Automatically assign ID using name of history item(s).
- Select analysis mode: Default settings only.
  - Do you want to use presets? No, just defaults.
- Do you want to tweak SAM/BAM Options? No
- Save Bowtie2 mapping statistics to the history? Yes.

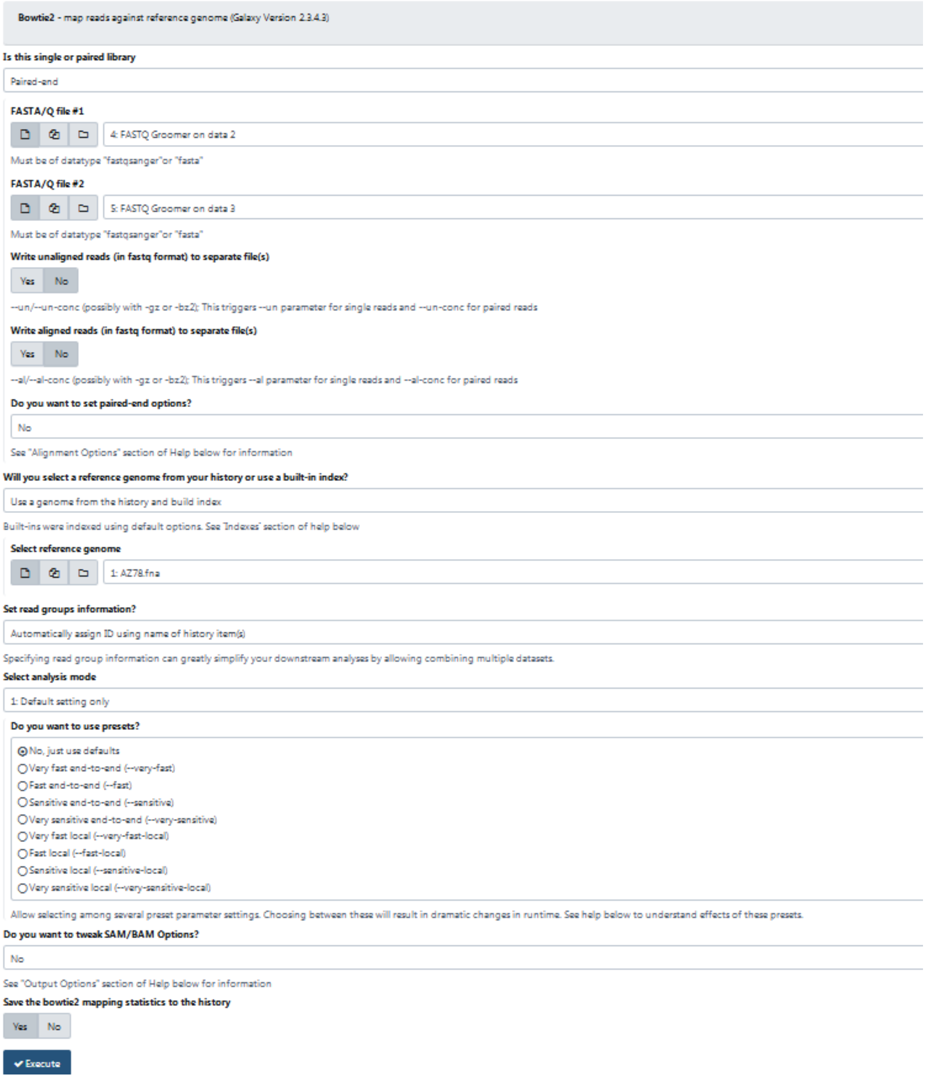

2.2. Assemble the unmapped reads.

Assemble the unmapped reads using SPAdes (8) (NGS: assembly>SPAdes). Apply the following settings:

- Single cell? No.
- Run only assembly? No.
- Careful correction? Yes.
- Automatically choose k-mer values Yes
- Coverage Curoff User specific
    - i. Coverage cutoff value 10
- Libraries are IonTorrent reads? No
- Libraries
    - i. Library type Paired-end/Single reads.
    - ii. Orientation ->-< (fr) make sure the reads are in this orientation).
    - iii. Files
        1. Select file format: separate input files.
            - a. Forward reads unmapped reads pair 1.
            - b. Reverse reads unmapped reads pair 2.
- Leave the remaining fields as set by default.

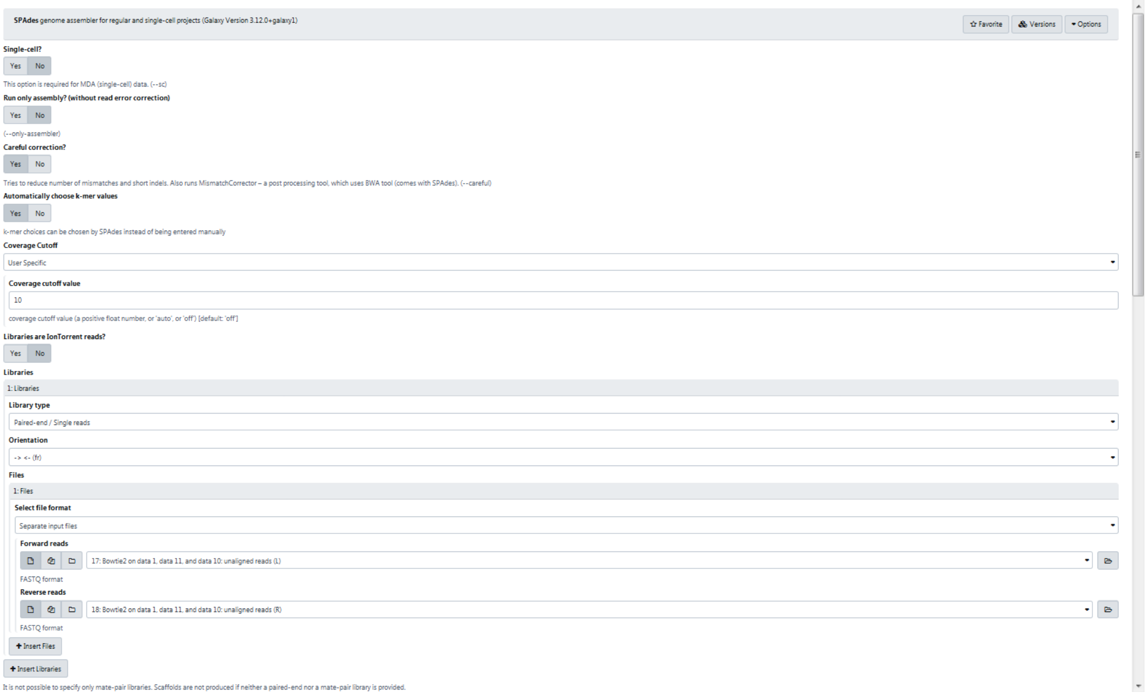

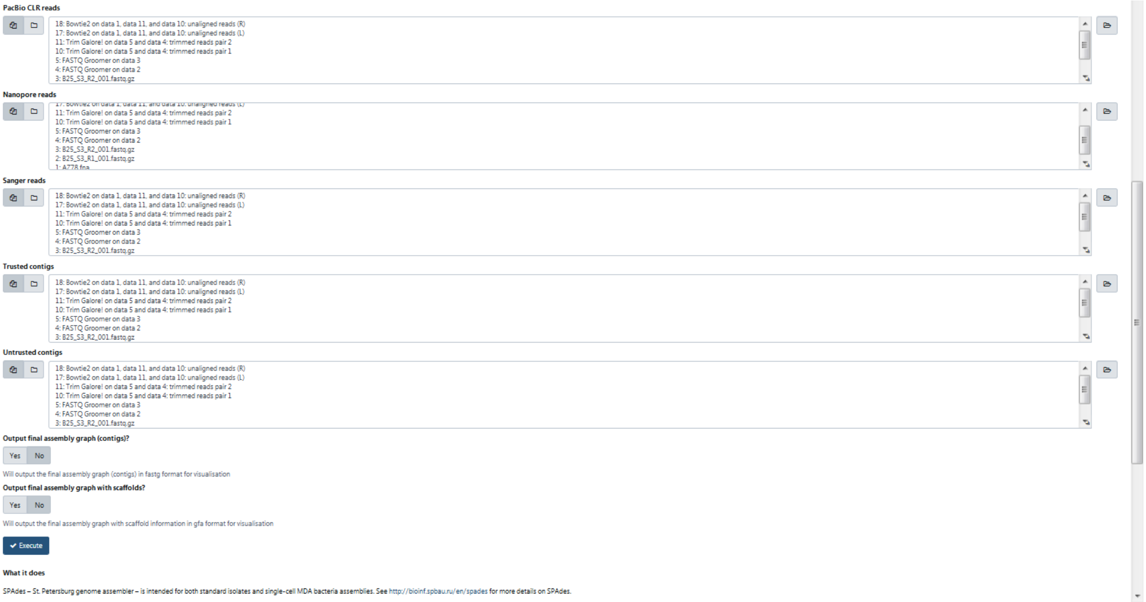

2.3. Filter assembled sequences by length.

Filter out contigs below a length and coverage cutoff (FASTA/FASTQ>Filter sequences by length).

- Fasta file: SPADes contigs (fasta).
- Minimal length: 200 (the minimum size for a scaffold in the GenBank)
- Maximum length: 0.

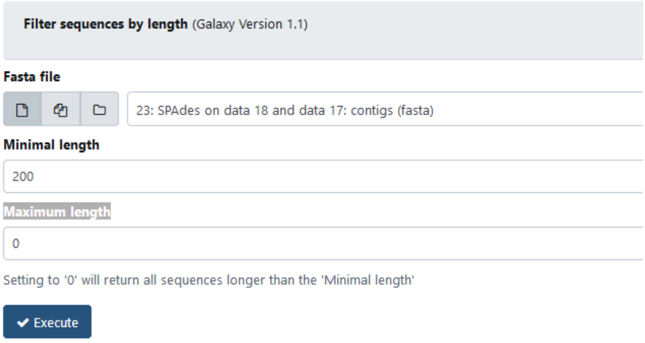

2.4. Obtain statistics for the assembled contigs (optional).

Obtain statistics for the filetered contigs (FASTA/FASTQ>Fasta statistics):

- Fast or multifasta file: Filtered sequences.

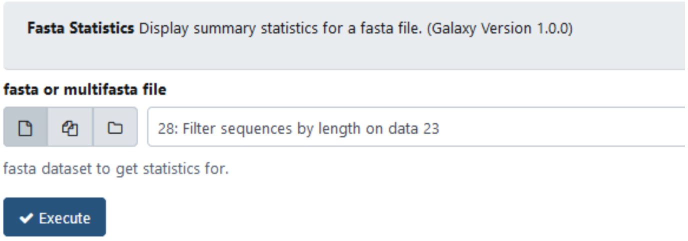

2.5. Extract ORFs.

Extract the ORFs from the contigs using the EMBOSS getorf tool (9, 10). (EMBOSS>getorf):

- Sequences: Filtered contigs (multi-fasta).
- Code to use: Bacterial.
- What to output: Nucleic sequences between START and STOP codons.
- All START codons to code for Methionine: No.
- Find ORFs in the reverse compliment: Yes.
- Number of flanking nucleotides to output: 0.
- Leave the remaining fields as set by default.

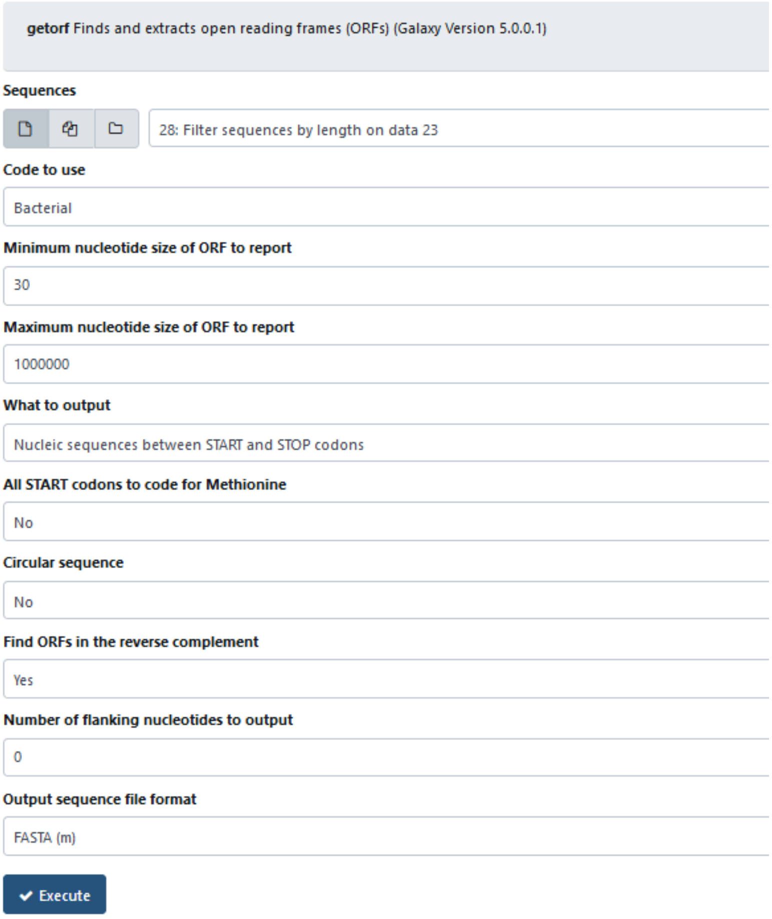

EMBOSS getorf produces sequences with troublesome characters in their headers. To prevent errors in subsequent steps one should delete these characters as follows:

Convert the multi-fasta file into tabular format (Convert Formats>Fasta-to-tabular).

- Convert these sequences: Select the file with the ORFs.
- How many columns to divide title string into? 1.
- How many title characters to keep? 0.

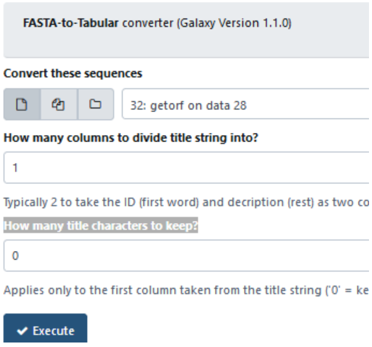

Replace the troublesome characters (Text Manipulation>replace text in a specific column)

- File to process: Select the file with the ORFs in tabular format.
- Replacement:
  - In column: 1.
  - Find pattern: \. (the backslash escapes the meaning of the dot as a special regular expression).
  - Replace with: -.
- Insert Replacement:
  - In column: 1.
  - Find pattern: (type a space).
  - Replace with: (leave untouched)

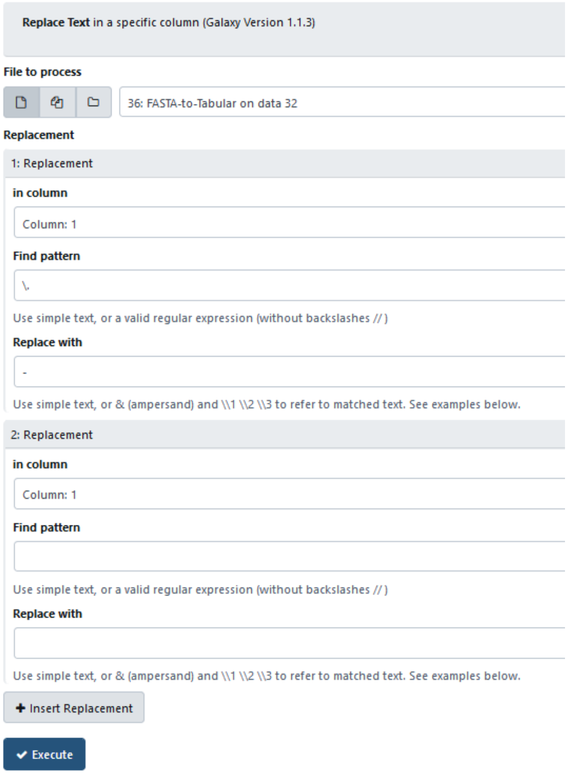

Convert the file back into fasta format (Conver formats>Tabular-to-FASTA).

- Tab-delimited file: Select the sequences in tabular format with the spaces deleted.
- Title column(s): Column 1.
- Sequences column: Column 2.

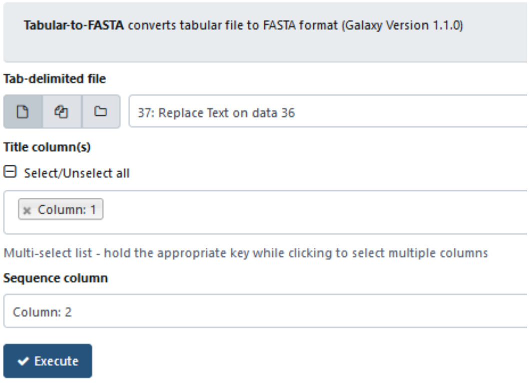

2.6. Discard ORFs with a BLAST hit.

2.6.1. Make a BLAST database.

Make a BLAST database (Blast +>NCBI BLAST + makeblastdb) applying the following settings:

- Molecule type of input: Nucleotide.
- Input FASTA files(s): Select the file with the nucleotide sequences.
- Title for BLAST database: Title of choice.
- Leave the remaining parameters as set by default.

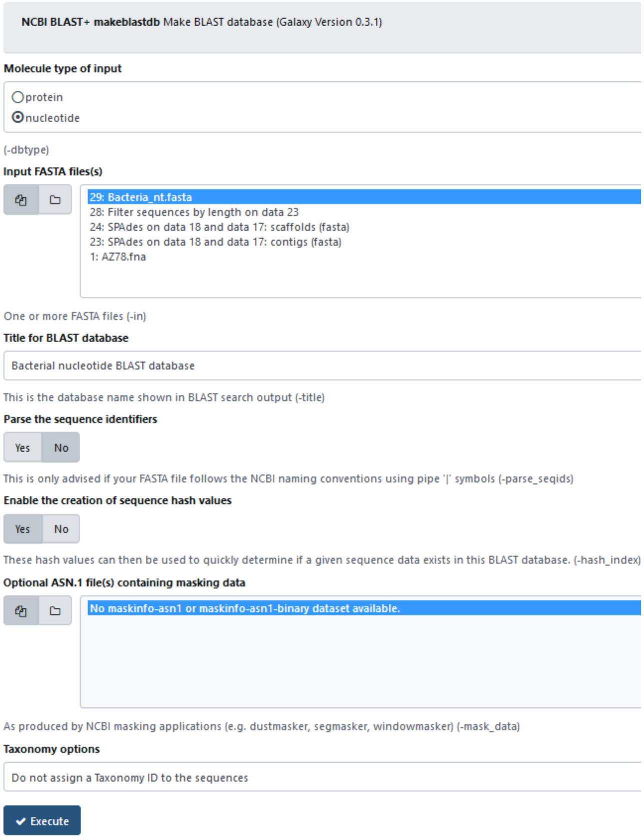

This step takes several hours.

2.6.2. Search homologues.

Run megablast (Blast +>NCBI BLAST + blastn) to find homologues to the extracted ORFs, with the following settings:

- Nucleotide query sequence(s): Select the filtered sequences.
- Subject database/sequences: Select the created BLAST database.
- Type of BLAST: megablast.
- Output format: Tabular (standard 12 columns).
- Advanced Options: Show advanced options.
    - Maximum hits to consider/show: 1.
    - Perform ungapped alignment only? Yes.

- Leave the remaining parameters as set by default.

Nucleotide query sequence(s)

32: getorf on data 28

(-query)

Subject database/sequences

BLAST database from your history

Nucleotide BLAST database

34: Bacterial nucleotide BLAST database

Type of BLAST

- (selected) megablast - Traditional megablast used to find very similar (e.g., intraspecies or closely related species) sequences
- blastn - Traditional BLASTN requiring an exact match of 11, for somewhat similar sequences
- blastn-short - BLASTN program optimized for sequences shorter than 50 bases
- dc-megablast - Discontiguous megablast used to find more distant (e.g., interspecies) sequences

(-task)

Set expectation value cutoff

0.001

(-evalue)

Output format

Tabular (standard 12 columns)

(-outfmt)

Advanced Options

Show Advanced Options

Filter out low complexity regions (with DUST)

Yes | No

(-dust)

Query strand(s) to search against database/subject

Both

(-strand)

Maximum hits to consider/show

1

Use zero for default limits. For HTML and plain text output this value is passed -num_descriptions and -num_alignments but f
internal limits during the search, which can in some cases exclude matches which would otherwise become the best hit.

Maximum number of HSPs (alignments) to keep for any single query-subject pair

The HSPs shown will be the best as judged by expect value. If this option is not set, BLAST shows all HSPs meeting the expect

Percent identity cutoff

0

Use zero for no cutoff (-perc_identity)

Word size for wordfinder algorithm

Leave blank for default, otherwise minimum 2 (-word_size)

Perform ungapped alignment only?

Yes | No

(-ungapped)

Should the query and subject defline(s) be parsed?

Yes | No

This affects the formatting of the query/subject ID strings (-parse_deflines)

Restrict search of database to a given set of ID's

No restriction, search the entire database

This feature provides a means to exclude ID's from a BLAST database search. The expectation values in the BLAST results are ba
FASTA file.

Minimum query coverage per hsp (percentage, 0 to 100)

0

See also the output column qcovhsp (-qcov_hsp_perc)

Multiple hits window size: use 0 to specify 1-hit algorithm, leave blank for default

Only relevant for dc-megablast, and otherwise ignored. Default window size changes with substitution matrix and BLAST type

Cost to open a gap

Leave blank for default. See tool help for defaults. (-gapopen)

Cost to extend a gap

Leave blank for default. See tool help for defaults. (-gapextend)

Execute

2.6.3. Discard the ORFs with a BLAST hit.

Cross-compare the starting ORFs with those with a BLAST hit (Join, Subtract, Group>Compare two Datasets) to obtain the ORFs with no BLAST hit.

- Compare: Select the file with the ORFs with the headers polished.
- Using column: Column 1.
- Against: select the output of the Blast search.
- And column: Column 1.
- To find: Non Matching rows of 1st dataset.

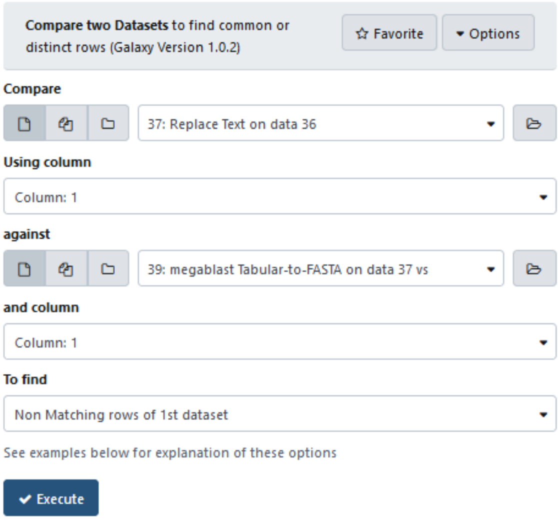

Download the file with the ORFs with no BLAST hit.

2.7. Obtain statistics for the strain-specific ORFs (optional).

Transform the file with the strain-specific ORFs into fasta format and obtain fasta file statistics as described previously.

3. Primer and probe design.

3.1. Design the primers.

Design primers for the strain-specific ORFs using BatchPrimer3 (12) from the USDA-ARS server (https://probes.pw.usda.gov/batchprimer3/). Note that the server only accepts files with <500 sequences so if the number of strain-specific ORFs surpasses this number the multi-fasta file must be split into smaller chunks. This can be done manually (e.g. converting the multi-fasta file to tabular in Galaxy, splitting the table in excel, and converting each part into fasta back in Galaxy), using the command line (there are many scripts available for this), or online (e.g. at the web site http://genomics.cicbiogune.es/SECRETOOL/Splitter.php). If the ORFs are split into multiple files, from this point on, one can continue using all sequences (i.e. all chunks) or just a fraction of them (e.g. the first 500).

Select the sequence file (the ORFs with no BLAST hit, or a subset of them) and apply the settings shown next (or any others that fit your needs):

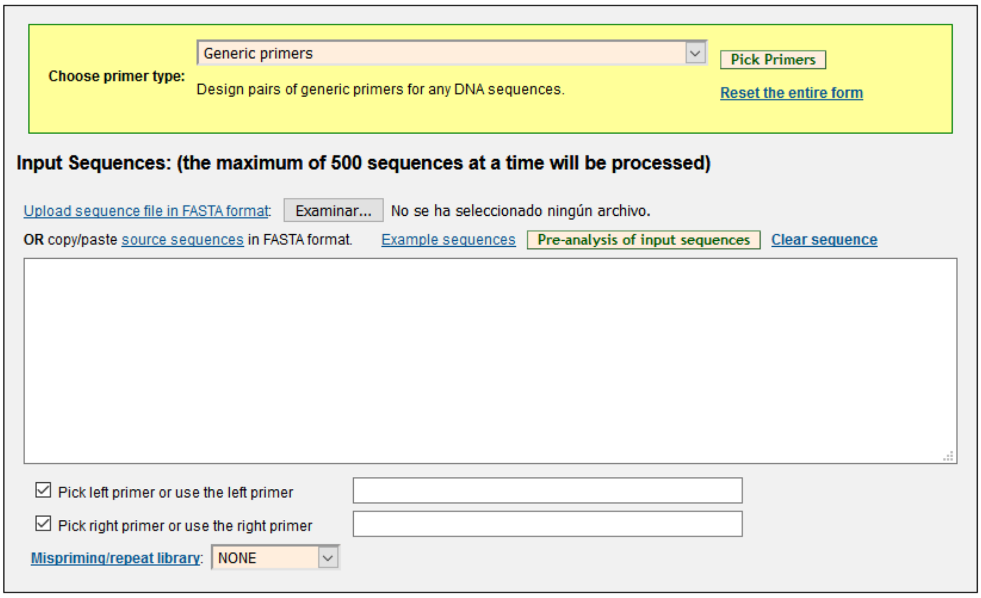

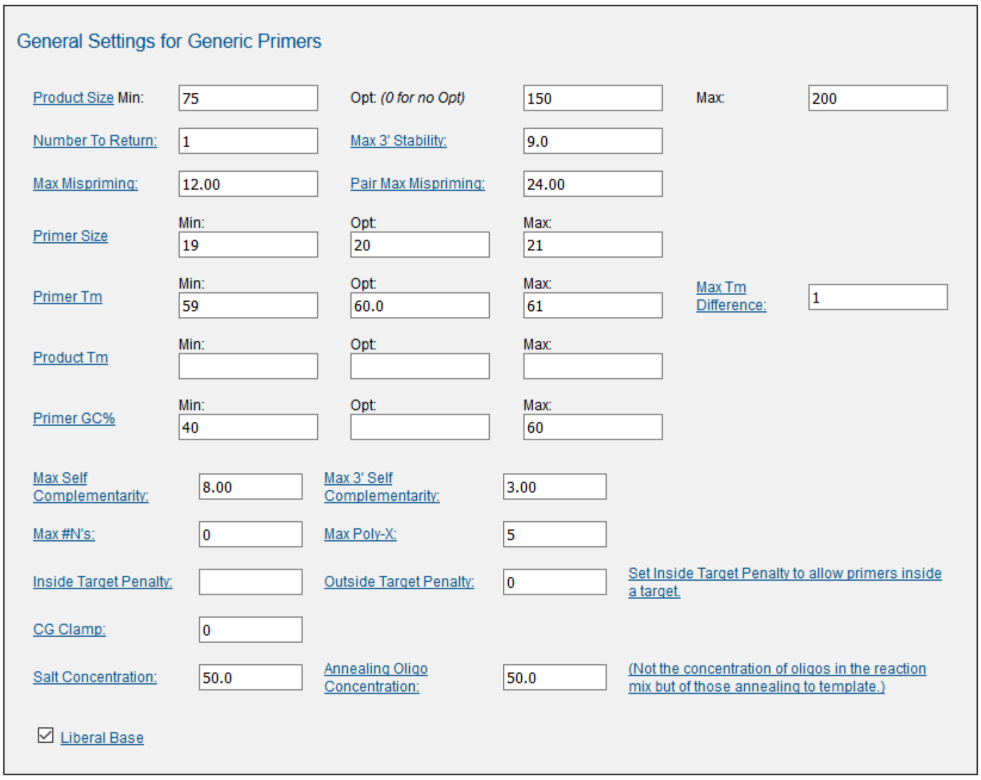

Leave the remaining settings as set by default and hit “pick primers”.

3.2. Obtain the amplicon sequences.

For this, Galaxy Australia and PrimerBlast are used in combination. For convenience, format grooming hereby is performed using both Excel and Galaxy, but the same results can be achieved in many other ways.

- Download the BatchPrimer3 results as an Excel spreadsheet and rearrange the primers so a primer pair is displayed in a single row:
    - Duplicate the data (copy/paste in the following columns).
    - Delete the first row of the duplicated cells so the reverse primers are in the same line as their respective forward primer
    - Select the first “Product size” column and from it, the blank cells (F5>Special>Blank)
    - Delete the rows in which the selected cells are (cells>delete).
    - Sort the sequences by the header.
- Calculate the amplicon coordinates, including the primers.
- Create a new excel spreadsheet with 3 columns: the sequence header, the amplicon start coordinate and the amplicon end coordinate; and save it as tabular-delimited txt.
- Upload this file to Galaxy Australia.
- Verify that the file is identified as bed (use the pencil icon in the history panel). If not, double check the files.
- Extract the amplicon sequences form the strain-specific ORFs using the coordinates (Fetch Sequences/Alignments>Extract Genomic DNA):
    - Fetch sequences for intervals in: Select the file with the amplicon coordinates.
    - Interpret features when possible: No.
    - Source of genomic data: History.
        - Using reference file: Select the file with the strain-specific ORFs.
    - Output data type: FASTA.

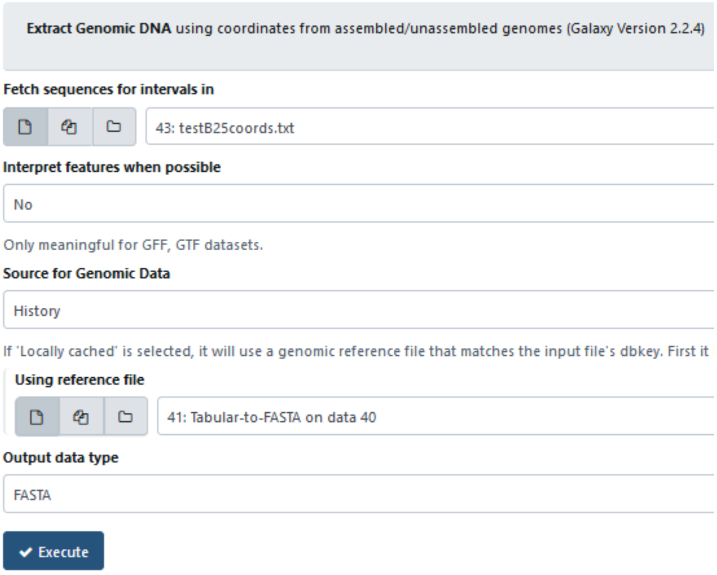

Note that the headers in the sequence file and those in the coordinate file must match for the program to associate the coordinates with the sequence.

- Transform the output fasta into tabular as described previously.
- Download the sequences.
- Paste the sequences in a new Excel spreadsheet.
- Delete the characters added to the sequence headers by the tool Extract Genomic DNA (tip: split text in columns separated by the underscore, delete the desired columns, and concatenate the columns again using the underscore as separator (use the & symbol).
- Paste the amplicon sequences next to their respective primer sequences and properties (verify that they follow the same order) to have the primer and amplicon sequences in the same row.
- Polish the spreadsheet so useless (index number, count, primer type, etc.) or repeated (Prod Size, Seq Length, Included Length, Pair any compl, Pair 3' compl) information is deleted.
- Sort the primer/probe sets according to the following criteria (in this order):

- 3’ compl. fwd primer 1 (fwd, forward primer).
- 3’compl. rev 2 (rev, reverse-complementary primer).
- Any compl. fwd.
- Any compl. rev.

3.3.Verify primer specificity *in silico*.

Run primer-BLAST (https://www.ncbi.nlm.nih.gov/tools/primer-blast/) on a selection of primer pairs, one by one, applying the following settings:

- Enter accession, gi, or FASTA sequence: paste the amplicon sequence.
- Use my own forward primer: paste the fwd primer.
- Use my own reverse primer: paste the rev primer.
- PCR product size: 75-200.
- Primer melting temperatures: 55/60/65/5 (max./opt/min/max. Tm diff.).
- Database: nr.
- Organism: bacteria (taxid:2).
- Show results in a new window: √
- Use new graphic view: √
- (Leave the remaining parameters as set by default).

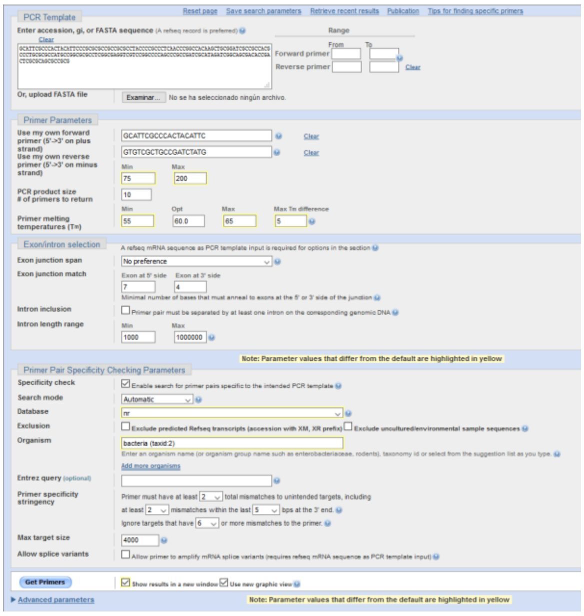

Discard all primer pairs that show an off-target in the selected database.

3.4. Design internal oligos.

Run Primer3Plus (13) online (http://www.bioinformatics.nl/cgi-bin/primer3plus/primer3plus.cgi/) with the following configuration:

- Paste source sequence below: paste the amplicon sequence excluding the primer sequences.
- Pick left Primer: uncheck; Paste the primers previously designed.
- Pick left Primer: uncheck; Paste the primers previously designed.
- Pick Internal Hybridization Oligo: √.
- Go to “Internal Oligo” tab:
    - Hyb Oligo Size → 18/20/22 (min/opt/max).
    - Hyb Oligo Tm → 67/70/73 (min/opt/max; ha de ser 10ºC>Tm primer).
    - Hyb Oligo GC% → 20/50/80 (min/opt/max).
- Leave the remaining patameters as set by default.

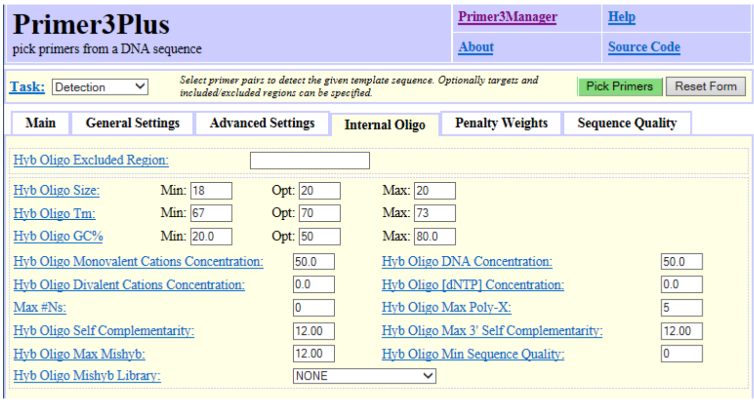

Discard all primer pairs that yield an amplicon that does not allow the design of an internal hybridization oligo.

Referencias

1. **Low L, Tammi M.** Bioinformatics doi:10.1142/10159.

2. **Afgan E, Baker D, Batut B, van den Beek M, Bouvier D, Čech M, Chilton J, Clements D, Coraor N, Grüning BA, Guerler A, Hillman-Jackson J, Hiltemann S, Jalili V, Rasche H, Soranzo N, Goecks J, Taylor J, Nekrutenko A, Blankenberg D.** 2018. The Galaxy platform for accessible, reproducible and collaborative biomedical analyses: 2018 update. Nucleic Acids Res **46:**W537-W544.

3. **Blankenberg D, Gordon A, Von Kuster G, Coraor N, Taylor J, Nekrutenko A.** 2010. Manipulation of FASTQ data with Galaxy. Bioinformatics **26:**1783-1785.

4. **Andrews S.** FastQC: A quality control tool for high throughput sequence data.

5. **Krueger F.** 2018. A wrapper tool around Cutadapt and FastQC to consistently apply quality and adapter trimming to FastQ files, with some extra functionality for MspI-digested RRBS-type (Reduced Representation Bisufite-Seq) libraries., https://www.bioinformatics.babraham.ac.uk/projects/trim_galore/.

6. **Langmead B, Salzberg SL.** 2012. Fast gapped-read alignment with Bowtie 2. Nat Meth **9:**357-359.

7. **Afgan E, Sloggett C, Goonasekera N, Makunin I, Benson D, Crowe M, Gladman S, Kowsar Y, Pheasant M, Horst R, Lonie A.** 2015. Genomics Virtual Laboratory: a practical bioinformatics workbench for the cloud. PLoS One **10:**e0140829.

8. **Bankevich A, Nurk S, Antipov D, Gurevich AA, Dvorkin M, Kulikov AS, Lesin VM, Nikolenko SI, Pham S, Prjibelski AD, Pyshkin AV, Sirotkin AV, Vyahhi N, Tesler G, Alekseyev MA, Pevzner PA.** 2012. SPAdes: a new genome assembly algorithm and its applications to single-cell sequencing. J Comput Biol **19:**455-477.

9. **Rice P, Longden I, Bleasby A.** 2000. EMBOSS: the European Molecular Biology Open Software Suite. Trends Genet **16:**276-277.

10. **Blankenberg D, Taylor J, Schenck I, He J, Zhang Y, Ghent M, Veeraraghavan N, Albert I, Miller W, Makova KD, Hardison RC, Nekrutenko A.** 2007. A framework for collaborative analysis of ENCODE data: making large-scale analyses biologist-friendly. Genome Res **17:**960-964.

11. **Zhang Z, Schwartz S, Wagner L, Miller W.** 2000. A greedy algorithm for aligning DNA sequences. J Comput Biol **7:**203-214.

12. **You FM, Huo N, Gu YQ, Luo MC, Ma Y, Hane D, Lazo GR, Dvorak J, Anderson OD.** 2008. BatchPrimer3: a high throughput web application for PCR and sequencing primer design. BMC Bioinformatics **9:**253.

13. **Untergasser A, Nijveen H, Rao X, Bisseling T, Geurts R, Leunissen JA.** 2007. Primer3Plus, an enhanced web interface to Primer3. Nucleic Acids Res **35:**W71-74.

14. **Pfaffl MW.** 2001. A new mathematical model for relative quantification in real-time RT-PCR. Nucleic Acids Res **29:**e45-e45.
