# Supplementary material for: Design of Bacterial Strain-Specific qPCR Assays Using NGS Data and Publicly Available Resources and Its Application to Track Biocontrol Strains
Source: Front Microbiol. 2020 Mar 10;11:208. doi: 10.3389/fmicb.2020.00208 (PMC7077341; doi:10.3389/fmicb.2020.00208)
Supplement: Supplementary file 2 [file Data_Sheet_2.pdf]

Supplemental Table 1. Primers and probes obtained used with the proposed workflow after *in silico* design, testing for technical viability and strain specificity.

mBCA	Primer	Primer sequence	Tm (°C)	Prod. len. (nt)	Probe sequence	Probe Tm (°C)	Labels (5'/3')
B24	2f	ATGGGGTGGAAGTCAAAGG	59.8	120	TCTCAACCATTGCTTCCGGTCA	57.3	FAM/ BHQ1
	2r	GGTAGTGCCGTGGAGGAATA	60.0				
	5f	GAGTGCGTCGGGTTGAGT	59.8	147	TGAGCGTTACCGTGCGTCCG	65.0	R610/ BHQ-2
	5r	CATAGTCGGGTCGCCTAGC	60.8				
	4f	CTACCCCTCGTATCGCTTCC	61.0	151	ACCTGCGCGTGGATCCGAAG	63.5	Cy5/ BBQ
	4r	ACGTCTCGCCTCTATCCTCA	60.0				
B25	473f	GTCAATATCCCGGTGGTCTG	60.2	74	AACCTGGCGGTGTTGCTGGG	63.5	FAM/ BHQ1
	473r	AGCGACAGATAGCCCACGTA	60.8				
	27f	CACTGGGGGTGATAGGTACG	60.3	77	ACCACCAACTGCCGGGTCGA	65.0	R610/ BHQ-2
	27r	GGAAACGACGAGCGTGTAAT	60.1				
	24f	CGCGGTACTTCACGTCATAA	59.8	59	CGGACCACGACGGGAGTCA	63.1	Cy5/ BBQ
	24r	TGAAGGAGTACGAGGGATGG	60.1				
B410	220f	GGAGGAAGTTCCGGTTCAAT	64.1	112	GGCAACCGTGAAAAAGCCTGCC	70.8	FAM/ BHQ1
	220r	TCTGAGATCAGTGCCTCGAA	63.8				
	167f	CATTGCTCCAACCTCTTGGT	64.0	101	CACCTCCCAGAGGACCTGAACG	67.6	R610/ BHQ-2
	167r	CTACTGGCACAACTGGAGCA	64.1				
	35f	ATATTTAGCCCGGCTTTGGT	63.3	102	GGTCGATTCGCGGCTCCTGA	70.2	Cy5/ BBQ
	35r	GACCTCTGCGAATGTTGTGA	64.0				
B2017	18f	ATCCGTCCAAAGGCGTGAG	67.5	355	ACGCGGCGCTGCAGGCAAAA	63.5	FAM/ BHQ1
	18r	GACTCTCCTTGTATGTAGGCCAT	62.2				
	20f	GTTCCCAGCACTGGAATGGA	67.4	833	GGCTTGGCCGCGATCGCAGT	65.5	R610/

	20r	TGGGACGATCCTTTTCCTGC	68.4				BHQ-2
	17f	TTGACCTTGGGCAGTTGGTT	66.6	865	CGGCCGATCATGTGCCGCGG	67.5	Cy5 BBQ
	17r	GGCGCTTCGTCAACATCATC	68.0				
B2021	43f	TTGTTCGGATCAGCGACTC	59.93	152	TGCCTCGATTGCGAAAATGCA	69.0	FAM BHQ1
	43r	GATGTCGCCACCTCGATAAT	59.92				
	2f	ATGGGGCTATCGTCGAGTC	60.1	150	CGCGCCATATTGCGGAAAA	60.0	R610 BHQ-2
	2r	AGGTTTCCGGAGGAAAGAAA	60.1				
	182f	CTCAAAAGCGGGGTCAGTTA	60.24	134	TTTGGCGTTGACACGGCTCG	69.8	Cy5 BBQ
	182r	TGGTCATCATCACGGAAATG	60.33				

BBQ650, BlackBerry® Quencher 650; BHQ-2, BlackHole® Quencher-2; mBCA, microbioal biocontrol agent.

Supplemental Table 2. Strains used to test primer pair and probe specificity.

Target	Off-target	Species (by 16S sequencing)
B24	B520	*Microbacterium foliorum*
	B1144	*Microbacterium testaceum/foliorum/oxydans*
	B1150	*Microbacterium sp./profundi/shaanxiense*
	B1539	*Microbacterium sp./schleiferi*
	B1780	*Microbacterium oxydans/maritypicum*
	B2014	*Microbacterium oxydans*
	B2015	*Microbacterium oxydans*
	B2044	*Microbacterium sp.*
	B2055	*Microbacterium sp.*
	B2058	*Microbacterium sp.*
	B2062	*Microbacterium aurum/pumilum/aoyamense*
	B2065	*Microbacterium arthrospaerae*
	B2078	*Microbacterium foliorum/oxydans/shrimpcida*
	B2118	*Microbacterium foliorum/oxydans*
	B2133	*Microbacterium sp.*
	B2433	*Microbacterium maritypicum/oxydans/Erwinia sp.*
	B2434	*Microbacterium sp.*
	B2451	*Microbacterium oxydans/maritypicum*
	B2507	*Microbacterium oxydans/maritypicum*
	B2538	*Microbacterium esteraromaticum*
	B2577	*Microbacterium phyllosphaerae*
	B2644	*Microbacterium esteraromaticum*
	B2649	*Microbacterium testaceum/foliorum/oxydans*
	B2652	*Microbacterium sp./chleiferi*
	B2653	*Microbacterium sp.*
	B2661	*Microbacterium hydrocarbonoxydans*
	B2718	*Microbacterium sp.*
B25	B322	*Lysobacter enzymogenes*
	B733	*Lysobacter capsici*
	B759	*Lysobacter antibioticus*
	B763	*Lysobacter antibioticus*
	B1380	*Lysobacter capsici*
	B2596*	*Lysobacter enzymogenes* C3
B410	B971	*Bacillus idriensis*
	B997	*Bacillus megaterium*
	B1013	*Bacillus marisflavi*
	B1080	*Bacillus sp./cereus*

	B1370	*Bacillus licheniformis*
	B1495	*Bacillus megaterium*
	B1563	*Bacillus simplex*
	B1883	*Bacillus simplex*
	B1912	*Bacillus licheniformis*
	B2198	*Bacillus subtilis*
	B2309	*Bacillus litoralis/niabensis/simplex)*
	B2361	*Bacillus licheniformis/aerius*
	B2399	*Bacillus litoralis*
	B2424	*Bacillus subtilis*
	B2525	*Bacillus niabensis*
	B2541	*Bacillus horikoshii*
	B2634	*Bacillus cereus/thruingiensis/wiedmannii*
B2017	47	*Pseudomonas psychrotolerans*
	86	*Pseudomonas chlorhoraphis*
	87	*Pseudomonas putida*
	194	*Pseudomonas chlororaphis*
	402	*Pseudomonas putida*
	678	*Pseudomonas resinovorans*
	767	*Pseudomonas sp./pseudoalcaligenes*
	770	*Pseudomonas resinovorans*
	807	*Pseudomonas plecoglossicida*
	898	*Pseudomonas moraviensis*
	951	*Pseudomonas sp./putida/fluorescens*
	1146	*Pseudomonas rhodesiae*
	1198	*Pseudomonas poae/trivialis*
	1269	*Pseudomonas fluorescents/monteilii/sp./putida*
	1295	*Pseudomonas lini/mediterranea*
	1308	*Pseudomonas frederiksbergensis*
	1404	*Pseudomonas frederiksbergensis*
	1410	*Pseudomonas putida*
	1449	*Pseudomonas putida*
	1453	*Pseudomonas putida/monteilii*
	1517	*Pseudomonas fulva*
	1927	*Pseudomonas fluorescens/marginalis/veronii*
	1968	*Pseudomonas plecoglossicida/putida/monteilii/taiwanensis*
	2016	*Pseudomonas fluorescens/koreensis*
	2018	*Pseudomonas putida*
	2019	*Pseudomonas sp./putida*

	2073	*Pseudomonas sp./fluorescens/putida*
	2081	*Pseudomonas koreensis/moraviensis/putida/clemancea*
	2104	*Pseudomonas mandeli*
	2105	*Pseudomonas putida/koreensis*
	2106	*Pseudomonas migulae*
	2107	*Pseudomonas punonensis*
	2138	*Pseudomonas orientalis*
	2178	*Pseudomonas sp./clemancea/moraviensis*
	2299	*Pseudomonas plecoglossicida/monteilli/putida*
	2346	*Pseudomonas putida/monteilii/taiwanensis/plecoglossicida*
	2347	*Pseudomonas sp./pseudoalcaligenes*
	2409	*Pseudomonas koreensis/moraviensis/putida/clemancea*
	2435	*Pseudomonas pseudoalcaligenes*
	2438**	*Pseudomonas putida* 90 (ATCC11607)
	2452	*Pseudomonas koreensis*
	2453	*Pseudomonas koreensis*
	2463	*Pseudomonas koreensis/moraviensis/putida/fluorescens*
	2473	*Pseudomonas koreensis/moraviensis/putida*
	2475	*Pseudomonas lurida/tolaasii*
	2517	*Pseudomonas putida/plecoglossicida*
	2518	*Pseudomonas putida/monteilii/taiwanensis/plecoglossicida/humanensis*
B2021	B393	*Pseudomonas azotoformans/putida/gessardii*
	B906	*Pseudomonas libanensis/azotoformans/fluorescens/poae/gessardii/reactans*
	B949	*Pseudomonas grimontii*
	B1133	*Pseudomonas grimontii*
	B1146	*Pseudomonas rhodesiae*
	B1176	*Pseudomonas veronii/marginalis/fluorescens*
	B1187	*Pseudomonas veronii/fluorescens*
	B1198	*Pseudomonas poae/trivialis*
	B1202	*Pseudomonas extremaustralis/marginalis/fluorescens/veronii/fulva*
	B1286	*Pseudomonas fluorescens*
	B1927	*Pseudomonas fluorescens/marginalis/veronii*
	B2138	*Pseudomonas orientalis*
	B2447	*Pseudomonas libanensis/azotoformans/fluorescens/poae/gessardii/reactans*
	B2454	*Pseudomonas libanensis/azotoformans/fluorescens/gesssardii/poae*
	B2467	*Pseudomonas libanensis/azotoformans/fluorescens/poae/gessardii*
	B2472	*Pseudomonas fluorescens/poae/extremorientalis*

	B2475	*Pseudomonas lurida/tolaasii*
	B2553	*Pseudomonas fluorescens*

* Kindly provided by Prof. Gary Yuen. ** Purchased from the Spanish Type Culture collection.

Supplemental Table 3. Physico-chemical parameters of waters.

	pH	Conductivity ($\mu S \cdot cm^{-1}$)	Turbidity (OD_{420})	Dry residue ($mg \cdot L^{-1}$)
St. Cugat well	8.04	2422	0.003	131.9
Anoia river	7.93	2750	0.014	138
Foix river	8.24	1326	0.015	68.6
Tap	7.7	853	0.008	36

The dry residue was measured by drying 0.1 L at 80 ºC for 24 h in a volumetric flask and weighting the full and dry flasks equilibrated to room temperature in an FV-120C scale (Gram). Further information about the water qualities can be obtained at http://www.ub.edu/barcelonarius/web/index.php (Anoia and Foix rivers) and https://www.aiguesdevilafranca.cat/es-es/El-agua-en-Vilafranca/Control-calidad-del-agua (tap water) Further information of the St. Cugat well water is not available.

Supplemental Table 4. qPCR efficiency using intercalating dye and hydrolysis probes under increasing multiplexing degree.

mBCA	Primer (label)	Intercalating dye	Hydrolysis probes		
			Monocolor	Dual color	Triplex
B24	2 (FAM)	1.971	1.968	1.953/2.012	1.991
	4 (R610)	2.230	1.896	1.978/2.041	1.875
	5 (CY5)	2.142	1.920	1.884/1.848	1.982
B25	24 (FAM)	1.868	1.987	2.029/1.909	2.040
	27 (R610)	1.938	1.982	2.047/1.987	2.000
	473 (CY5)	1.956	2.045	1.921/1.964	2.010
B410	35 (FAM)	2.288	2.376	1.774/1.829	1.978
	20 (R610)	2.109	1.945	1.895/1.848	1.834
	167 (CY5)	2.176	1.828	1.820/1.839	1.835
B2017	18 (FAM)	1.800	1.635	1.606/1.712	1.686
	20 (R610)	1.630	2.005	1.996/2.009	1.994
	17 (CY5)	1.889	1.919	1.959/1.912	1.924
B2021	43 (FAM)	1.921	1.945	1.863/2.183	2.455
	182 (R610)	2.083	1.95	2.070/2.035	2.16
	2 (CY5)	2.637	2.213	2.117/2.126	2.060

Supplemental Table 5. Absolute limits of detection of 5 bacterial biocontrol strains using pure amplicon as PCR template.

Strain	Limit of detection (cells μL^{-1} template)	Linearity (orders of magnitude)
B24	35	7
B25	1	7
B410	122	7
B2017	114	5
B2021	12.5	5
